# Supplementary material for: Women’s health in The BMJ: a data science history
Source: BMJ Open. 2020 Oct 21;10(10):e039759. doi: 10.1136/bmjopen-2020-039759 (PMC7580070; doi:10.1136/bmjopen-2020-039759)
Supplement: Author's manuscript [file bmjopen-2020-039759.draft_revisions.pdf]

BMJ Open is committed to open peer review. As part of this commitment we make the peer review history of every article we publish publicly available.

When an article is published we post the peer reviewers' comments and the authors' responses online. We also post the versions of the paper that were used during peer review. These are the versions that the peer review comments apply to.

The versions of the paper that follow are the versions that were submitted during the peer review process. They are not the versions of record or the final published versions. They should not be cited or distributed as the published version of this manuscript.

BMJ Open is an open access journal and the full, final, typeset and author-corrected version of record of the manuscript is available on our site with no access controls, subscription charges or pay-per-view fees (http://bmjopen.bmj.com).

If you have any questions on BMJ Open's open peer review process please email info.bmjopen@bmj.com

BMJ Open

Women's Health in *The BMJ*: A Data Science History

Journal:	*BMJ Open*
Manuscript ID	bmjopen-2020-039759
Article Type:	Original research
Date Submitted by the Author:	24-Apr-2020
Complete List of Authors:	Hamulyák, Eva; Amsterdam UMC - Locatie AMC, Department of Vascular Medicine<br>Brockmeier, Austin; University of Delaware, Department of Electrical & Computer Engineering<br>Killas, Johanna; University of Toronto, Health Studies Programme<br>Ananiadou, S; The University of Manchester National Centre for Text Mining, Department of Computer Science; The Alan Turing Institute<br>Middeldorp, Saskia; Amsterdam UMC - Locatie AMC, Department of Vascular Medicine<br>Leroi, Armand; Imperial College London, Department of Life Sciences; Imperial College London, Data Science Institute
Keywords:	MEDICAL HISTORY, MEDICAL JOURNALISM, QUALITATIVE RESEARCH, STATISTICS & RESEARCH METHODS

SCHOLARONE™
Manuscripts

I, the Submitting Author has the right to grant and does grant on behalf of all authors of the Work (as defined in the below author licence), an exclusive licence and/or a non-exclusive licence for contributions from authors who are: i) UK Crown employees; ii) where BMJ has agreed a CC-BY licence shall apply, and/or iii) in accordance with the terms applicable for US Federal Government officers or employees acting as part of their official duties; on a worldwide, perpetual, irrevocable, royalty-free basis to BMJ Publishing Group Ltd ("BMJ") its licensees and where the relevant Journal is co-owned by BMJ to the co-owners of the Journal, to publish the Work in this journal and any other BMJ products and to exploit all rights, as set out in our licence.

The Submitting Author accepts and understands that any supply made under these terms is made by BMJ to the Submitting Author unless you are acting as an employee on behalf of your employer or a postgraduate student of an affiliated institution which is paying any applicable article publishing charge ("APC") for Open Access articles. Where the Submitting Author wishes to make the Work available on an Open Access basis (and intends to pay the relevant APC), the terms of reuse of such Open Access shall be governed by a Creative Commons licence – details of these licences and which Creative Commons licence will apply to this Work are set out in our licence referred to above.

Other than as permitted in any relevant BMJ Author's Self Archiving Policies, I confirm this Work has not been accepted for publication elsewhere, is not being considered for publication elsewhere and does not duplicate material already published. I confirm all authors consent to publication of this Work and authorise the granting of this licence.

For peer review only

Women's Health in *The BMJ*: A Data Science History

Eva N. Hamulyák[1*], Austin J. Brockmeier[2], Johanna D. Killas[3], Sophia Ananiadou[4,5], Saskia Middeldorp[1†], Armand M. Leroi[6,7§]

[1]*Department of Vascular Medicine, Amsterdam Cardiovascular Sciences, Amsterdam UMC, University of Amsterdam, Amsterdam, the Netherlands*
[2]*Department of Electrical & Computer Engineering, University of Delaware, Newark, Delaware, USA*
[3]*Health Studies Programme, University of Toronto, Toronto, Canada*
[4]*National Centre for Text Mining, Department of Computer Science, University of Manchester, Manchester M1 7DN, UK*
[5]*The Alan Turing Institute, London NW1 2DB, UK*
[6]*Department of Life Sciences, Imperial College London, South Kensington Campus, London SW7 2AZ, UK*
[7]*Data Science Institute, Imperial College London, South Kensington Campus, London SW7 2AZ, UK*

[*]Corresponding author, [†]joint senior author, [§]corresponding author, joint senior author

Correspondence
Eva N. Hamulyák
E-mail: e.n.hamulyak@amsterdamumc.nl
Tel: +31 20 566 7516

Armand M. Leroi
E-mail: a.leroi@imperial.ac.uk
Tel: +44 20 7594 2396

Word count: 3648

Word count abstract: 240

Figures: 5

References: 65

Keywords: Women's health – *BMJ* – clinical studies – representation - editor

Abstract

Objective
To determine how the representation of women's health has changed in clinical studies over the course of seventy years.

Design
Observational study of 71,866 research articles published between 1948 and 2018 in *The BMJ*.

Main outcome measures
The incidence of women-specific health topics over time. General linear, additive, and segmented regression models were used to estimate trends.

Results
Over seventy years, the overall odds that a word in a *BMJ* research article was "woman" or "women" increased by an annual factor of 1.023, but this rate of increase varied by clinical specialty with some showing little or no change. The odds that an article was about some aspect of women-specific health increased much more slowly, by an annual factor of 1.004. The incidence of articles about particular areas of women-specific medicine such as pregnancy did not show a general increase, but rather fluctuated over time. The incidence of articles making any mention of women, gender or sex declined between 1948 and 2005, after which it rose steeply so that by 2018 few papers made no mention of them at all.

Conclusions
Over time women have become ever more prominent in *BMJ* research articles. However, the importance of women-specific health topics has waxed and waned as researchers responded ephemerally to medical advances, public health programmes, and socio-legal changes. The appointment of a woman editor-in-chief in 2005 may have had a dramatic effect on whether women were mentioned in research articles.

Keywords
Women's health – *BMJ* – clinical studies – representation - editor

Strengths and limitations of this study

- This is the first large-scale text-mining study that investigates how women have been represented in the clinical literature.
- Our study is about modern medicine, uses text mining tools to quantify the content of thousands of clinical articles, and inferential statistics to test hypotheses.
- Our study is based on a single journal, *The BMJ*, one that strongly reflects British concerns.
- Taking into account that topic analysis is a rather blunt instrument for discovering what articles are about, there is a general tendency for women to become increasingly prominent in the clinical literature over time.
- Statistical patterns of historical change are difficult to interpret in causal terms, but should be of interest to historians who can investigate their causes using traditional methods.

Introduction

Sex matters in medicine [1]. Not just because women and men differ in their reproductive biology, but because they also differ in the risks of many non-reproductive diseases such as auto-immune disorders and venous thromboembolism [2, 3], the relative importance of disease risk factors [4-6], rates of diagnosis, prognoses, and how they respond to drugs [7, 8].

Compared to men, women have historically been and, in some ways still are, ill-served by medicine [5]. The deficiencies of early medicine in the treatment of women are well documented [9–12], and so are the twentieth century's campaigns for change [13–17]. The history of women's health in modern — post-1900 — clinical science has, however, been little studied. An exception is the fraught discussion over whether women have been adequately represented, or properly studied, in clinical trials [18–23]. But the question of how women have been studied by clinical science is a much larger one than this. And its answer has consequences for health policy [1].

Here we apply text-mining techniques [24-30] to 71,866 articles published in *The BMJ* between 1948 and 2018 in order to find out what they say about women's health. We use the results of this analysis to provide a quantitative picture of the history of women's health, as seen through the lens of one journal, over the course of seventy years.

Materials & Methods

Constructing a corpus of *BMJ* research articles

The full text *BMJ* corpus was constructed to explore the recent history of medicine [26]. The text was obtained by means of optical character recognition (OCR) on articles that were published from 1840 onwards and subsequently scanned. The meta-data and PDFs are made publicly accessible by the U.S. National Library of Medicine and PubMed Central (PMC). PMC records were used to verify the meta-data and retrieve electronic versions of full text starting from 2009. To ensure OCR quality, we used a subset of articles from 01.01.1948 to 18.07.2018 that had a DOI entry in the PMC database, were published in *The BMJ*, including the clinical research edition, and had a publication type of "letter", "article-commentary", "review-article", or "research-article" [27]. Applying this criterion to 208,194 scanned articles and 2,151 electronic articles resulted in 74,937 articles that had been published over the course of approximately 70 years. **Figure 1** shows how these 74,937 research articles were filtered further to remove 1330 duplicate titles, 364 articles with fewer than 50 words, and 749 articles not present in both the word count and the topic probability datasets. In addition, we removed 628 articles published between 04.01.1997 and 14.02.1998 that were implausibly long, apparently due to parsing errors. The remaining 71,866 articles form the basis of our analysis.

Topic modelling

We pre-processed the corpus by removing 571 common English words, stopwords using the Smart system [31], and words which contained digits or hyphenated parts with less than one letter. To understand the content of the articles we used a text mining technique called topic modelling. The variant we used was Bayesian latent Dirichlet allocation (LDA) [32] implemented in the mallet-2.0.8 package [33]. The LDA algorithm estimates the probability of each word belonging to a topic and the probability of finding each topic in each article. This captures the likelihood of words appearing in the same document, but does not account for sentences, word order, capitalization, or punctuation. The number of topics, k, is set by the investigators; after some experimentation with $100 \leq k \leq 1000$, we settled on $k = 400$. The Bayesian hyper-parameters that control the dispersion of topics per article and words per topic were set at $\alpha = 1/300$ and $\beta = 1/100$; the algorithm optimized these every 10 epochs.

To aid the interpretation of our topics, a medical doctor (EH) labelled them based on the 20 most probable words associated with each (see online data). Each topic was subsequently assigned to a group of related topics designated as a "super-topic". For example, the super-topic "women breast cancer" contains three subsidiary topics: breast cancer, breast cysts (associated with cancer), and breast cancer screening. Since we were only interested in clinical medicine, we ignored the topics of health care management, the clinical literature, medical profession and regions. Of the 342 remaining topics that we used, 21 were clearly about women-specific health issues, such as pregnancy, oral contraceptives and breast cancer. Other topics besides these contained the word "women" in their most-probable words, but since they also included the word "men" they were not taken to be about women's health *per se*.

Estimating the incidence of topics and words

Our LDA model produced a probability, p_{ia}, that the ith topic is found in the ath article. To simplify analysis topic probabilities were discretized, labelling a topic as present if it exceeds a threshold probability [28]. Specifically, we identified a set of topics of interest for a given analysis (e.g., all clinical topics, or all medical condition topics), standardized the topic probabilities, p_{ia} of each article by the summed probabilities of the topics in that set, and then discretized them by assuming that an article is "about" some topic if $p_{ia} \geq 0.05$. Analogously, an article is about a given super-topic if the summed probabilities of the subsidiary topics ≥ 0.05. Having obtained counts of all topics of interest in all papers, we estimated the incidence rate of a topic as:

$$I_{it} = \frac{N_{it}}{N_t}$$

where N_{it} is the number of papers published at time t that are about the ith topic, and N_t is the total number of topics of interest identified in all papers published at that time. Note that, since papers may be about multiple topics, the denominator is not the total number of papers published in a given year.

In the same way, we estimate the incidence of a word as the number of times it occurs relative to the count of all words in all articles published at time *t*.

Constructing and analysing sub-corpora

Here we describe how we classified our articles into sub-corpora.

Articles about women-specific health topics and not

We classified articles into those that are about any aspect of women-specific health and those that are not. To do this we first filtered our dataset for the 342 clinical topics. Then, for each article, we summed probabilities of all 21 women's health-specific topics, as well the summed probabilities of all the other topics. We then standardized these two probabilities by their sum, and applied the $p_t \geq 0.05$ threshold to the standardized probabilities. These steps identified 10,158 papers about women-specific health.

Articles that are gender-vocal and gender silent

We classified articles into those that are gender vocal and gender silent. To do this we searched all articles for words that are related with gender or sexual differentiation: "woman/en", "female", "gender" and "sex", and labelled articles that used any of these words "gender-vocal", and those that used none of them "gender-silent". We validated our classification by examining the full text of 100 articles — 50 chosen at random from each group — and found that nearly all gender-vocal articles indeed studied women or girls. Gender silent articles, however, may study women or girls, but just not discuss them. These steps identified 28,837 gender-vocal articles.

Statistical analysis

All analyses were done in R. To model word or topic incidence as a function of time, we used a binomial generalized linear model (GLM) implemented in base R or a generalized additive model (GAM) implemented in the mcgv package. In order to identify breaks in individual series we carried out general linear regressions using the binomial family implemented in the segmented package [34] to estimate breakpoints. In all analyses we modelled time using publication date rather than year; for clarity, however, we show estimates of yearly incidence.

Patient and public involvement

No patients were involved in setting the research question or the outcome measures, nor were they involved in the design and implementation of the study.

Results

The rise of "women" 1948-2018

To discover how the representation of women has changed over time in *The BMJ* we began by examining word counts. Considering all articles, the words "woman" or "women" appeared about 3 times in every thousand words (0.27%), however, this incidence increased from about 1 in a thousand in 1948 to about 6 in 2018 (**Figure 2A**). Using a general linear model, we estimated that the odds of a word being "woman/en" increased by an annual factor of 1.023 ± 0.0003 (estimate ± 95% C.I.); $P < 2.0 \cdot e^{-16}$, faster than 81% of the 1000 most frequent words of clinical relevance (**Figure 2B**). By contrast, the odds of a word being "man/en" increased by 1.002 ± 0.0004; $P < 2.0 \cdot e^{-16}$) — about an order of magnitude slower.

We next asked whether the representation of women increased at the same rate in the articles of all clinical specialties. In fact, it's implausible that they should. This is because some specialties embrace important women-specific health conditions whose importance has varied over time. For example, the discovery in the 1960s that oral contraceptives and, later, hormonal replacement therapy, are risk factors for venous thrombosis resulted in many studies, quite a few of which were published in *The BMJ* [35–44]. In our scheme, they ephemerally increase the representation of women in articles about "haematology".

Here, however, we sought to exclude such effects. Instead, we wanted to study the frequency of occurrence of the words "woman" and "women" in articles that could, at least in principle, be about both sexes. For this reason, we first identified and excluded all articles about women-specific health topics (see below), which left us with 62,109, the bulk of the original sample. We then classified these articles into 20 clinical specialties using our super-topics (e.g., "cardiology", 22 topics), and estimated the incidence of the words "woman/en" in each using general linear models, as above. **Figure 2C** shows that the rate of increase of "woman/en" varied considerably among these specialties. While the odds of a word being "woman/en" increased by an annual factor of >1.02 in articles about nephrology, endocrinology and cardiology, many specialties had very low rates of increase (e.g., pharmacology) or were indistinguishable from zero (e.g, anaesthesiology). One specialty, psychiatry, actually declined.

The slow increase of women-specific health

Merely counting how often the words "woman/en" were mentioned in *BMJ* articles does not, however, tell us what they are about. Topic analysis does. Of the 400 topics discovered by the LDA model, 21were clearly about health issues specific to women. To capture whether an article is about any aspect of women-specific health we aggregated them into a single "women-specific health" super-topic. In 1948 the incidence of this supertopic was 11%; in 2018, 14%. The odds, then, that an article

was about any aspect of women-specific health increased by an annual factor of 1.004±0.0013; P = $9.28 \cdot e^{-10}$ (**Figure 3A**).

This seems surprisingly slow. By way of comparison consider two other super-topics: "clinical trial reports" (8 topics), which captures articles about randomized control trials, and "clinical case reports" (5 topics), which captures case studies. The odds that an article was about the first increased by an annual factor of 1.06±0.0018; P < $2.0 \cdot e^{-16}$, and about the second decreased by an annual factor of 0.97±0.0008; P < $2.0 \cdot e^{-16}$ (**Figure 3B**). These super-topics tell the great story of 20th century clinical science: its transformation from case-based observations to testing hypotheses in groups of patients. The increase of women-specific health is a minor phenomenon compared to that — at least it is so as seen through the lens of *The BMJ.*

Why was the increase in articles about women-specific health so slow? To investigate this, we examined the subsidiary topics (**Figure 4**). For clarity we grouped them into nine super-topics: "pregnancy" (8 topics),"neonatology" (2), "fertility" (2, excluding male fertility), "contraception" (1), "abortion" (1), "hormonal therapy" (1), "osteoporosis" (1), "'breast cancer" (3), and "cervical cancer" (2). The most common women-specific super-topic was "pregnancy" (4,744 articles); the least "hormonal therapy" (319 articles). Fits of general additive models show that the historical dynamics of these super-topics are characterized by fluctuations in incidence rather than a general increase. Many of the largest fluctuations are easily explained. For example, the increase in studies about contraception after 1960 is associated with the introduction of oral contraceptives [45] (**Figure 4D**). The UK's Abortion Act of 1967 [46], engendered much discussion about that topic (**Figure 4E**). The 1983 discovery that the human papillomavirus's (HPV) caused cervical cancer prompted a surge of articles about that topic, as did the implementation of an HPV vaccine in 2006 (**Figure 4I**). The introduction of a breast screening programme by NHS in 1988 resulted in many articles discussing its efficacy (**Figure 4H**). Not all changes are so easily explained: the increase in articles about pregnancy that occurred around 2005 does not appear to be driven by any obvious medical breakthrough (**Figure 4A**). But, in general, it appears that the history of women-specific health reflects the impact of contingent events such as medical advances, public health programmes, and socio-legal changes. Such events result in a flurry of articles which lasts for some years but then fades away as researchers turn to studying something else.

It took a woman?

Besides estimating the incidence of the words "woman/en", and the incidence of women's health-specific topics, in our articles, we also investigated how many of them mentioned women at all. To do this we classified our articles into "gender-vocal" articles that discuss women — or at least mention gender and sex differences in some way — and "gender-silent" articles that are oblivious to them. We found that in 1948 around 40% (240/606) of articles were "gender vocal", but that in 2018 84% (68/81) were (**Figure 5**). In order to confirm our results, we read the full text of many "gender silent"

papers published since 2010. We found some of them did, in fact, allude to women or sex or gender differences — but only in the data tables that we removed in pre-processing. This was particularly true of dozens of articles reporting clinical trials. Although these articles may have included women in their study samples, they were truly "gender silent" in that they did not analyse or report or discuss sex-specific differences in any way.

The increase in "gender vocal" papers did not occur gradually but rather suddenly, around 2005. A segmented GLM identified a single break at 2005 ± 0.45 years (95% confidence interval), before which the odds of an article being gender-vocal had declined gradually by an annual factor of 0.99 ± 0.0008; after which it increased by annual factor of 2.18 ± 0.015. What was the cause of this dramatic change? One possibility is that it was due to a change in editorial policy. In March 2005 Fiona Godlee succeeded Kamran Abbas (acting, 2004–2005) and Richard Smith (1991–2004) to become the first female editor-in-chief in the journal's 180-year history. We estimate that an article published by her was about 31% more likely to contain the words "woman/en" than one published by her immediate predecessors: Smith/Abbas: 0.0035 ± 0.00003; Godlee: 0.0046 ± 0.00005; odds-ratio: 1.308 ± 0.2949; P< 2 e^{-16}. Similarly, an article published by her was 45% more likely to be gender-vocal than one published by her immediate predecessors: Smith/Abbas: 0.37 ± 0.007; Godlee: 0.54 ± 0.014; odds-ratio: 2.04 ± 0.13; P< 2 e^{-16}. We did not detect a difference between the editors in the probability that an article is about women's health: Smith/Abbas: 0.0361 ± 0.0012; Godlee: 0.0365 ± 0.0023; odds-ratio: 1.0134 ± 0.0758; P=0.726). Thus, our data are consistent with the idea that Fiona Godlee had a substantial impact on the probability that a newly published article at least mentions women, but not on the probability that it considers some aspect of women-specific health.

Principal findings

We measured the representation of women in research articles in several ways. Two of these — the incidence of the words "woman/en" relative to all words (**Figure 2A**), and the incidence of gender-vocal articles (**Figure 5**) — have quite different dynamics, but concur in showing that women are now much more likely to be discussed in research articles than in the past. For at least fifty years women have asked that clinical science treat them equally to men [13–15, 47]. Clinical science, it seems, has responded — at least as seen in the pages of this journal, but the response has been uneven (**Figure 2C**). When we excluded articles about health issues specific to women, and estimated the incidence of the words "woman/en" in those that remained, we found that some — notably psychiatry — mentioned them relatively rarely; and that the rate at which they did so has not increased over seventy years. It is not that psychiatry has particularly many women-specific papers. We excluded only 9.4% (615/6595) of psychiatry articles, and our topic analysis did not identify a single women-specific psychiatric topic such as post-natal depression. This contrasts with oncology in which we excluded 21.8% (1026/4711) of articles, mostly about breast and cervical cancer; nevertheless, the rate at which women were mentioned in the remaining articles improved substantially. Cardiologists, too, appear to be addressing their "problem women" — as a *Lancet* editorial termed them [48] — but psychiatrists, it seems, must try harder [49–51]. Some are. The Royal College of Psychiatrists has had a "Women and Mental Health" special interest group since 1995.

Turning to articles about health issues specific to women, we found that their aggregated incidence has only increased very slightly over seventy years (**Figure 3A**). Examination of the subsidiary super-topics suggest that the dynamics of articles about women's health is driven less by a general trend towards increased representation, than by contingent medical advances, public health programmes, and socio-legal events. We make no general claims as to whether or not women-specific health is, or has been, adequately represented in the journal's pages. But we can identify some absences. Topic analysis does so since it is an unsupervised machine learning method that depends on the co-association of words among documents and, if a disease does not appear as a topic, that is probably because articles about it are rare. We have already mentioned one missing topic: post-natal depression. Endometriosis is another — even though it afflicts 1 in 10 women in their reproductive years [52]. Its relative invisibility is confirmed by simple word searches: "pregnancy", "breast", "ovarian" appear in 366, 270, and 63 of 2305 articles published since 2008, but "endometriosis" in only 15. These results are consistent with claims that the disorder has been neglected by clinical researchers and poorly understood by physicians [53–55]. Similarly, although women are far more likely to be the victims of domestic abuse than men [56], its medical consequences did not appear as a topic in any form. By contrast, "head injuries", which is mostly about concussions in rugby players, did. Such arguments cut both ways. The UK diagnosis and mortality rates of breast and prostate

cancer are nearly the same [57], but where breast cancer gets three topics ("breast cancer", "breast cysts", and "breast cancer treatment"), prostate cancer gets none. Word counts, however, show that the representation of these diseases is rapidly becoming more even (data not shown). Were we to repeat the analysis a decade hence, prostate cancer would likely have a topic of its own.

Our most intriguing result is the revelation that the incidence of gender-vocal articles increased dramatically around 2005, just when Fiona Godlee became editor-in-chief. It is easy to see how the two events might be causally connected. Perhaps authors, aware that *The BMJ* had acquired a new female editor, made sure to discuss the implications of their results in the context of women's health while previously they had been less assiduous in doing so. Or perhaps Godlee just made sure they did. Our results also suggest that her editorial intervention, if it existed, was limited, for we find no evidence that her editorship had an impact on whether or not an article was about a women-specific health topic. In order to clarify the interpretation of these results, we have written to Fiona Godlee asking her to comment on them.

The effect that women editors-in-chiefs might have on the careers of other women has been much discussed [58-63]. As far as we know their effect on the articles that their journals publish has not. In the absence of direct evidence, we cannot exclude the possibility that the sudden rise of gender-vocality at the *The BMJ* is due a change in the sensibilities of its authors quite independent of its editor. Even if there is a causal association, we cannot say how general it might be. But we could, in principle, find out. Several other important medical journals —*The Lancet*, *JAMA*, *JAMA Internal Medicine*, *Annals of Internal Medicine*, and the *Cochrane Library* among others — currently have, or have had, women editors-in-chief. Given the full texts of all their articles, it would not be difficult to apply our methods to those journals — as well as to the many that have never had one.

Strengths and limitations of this study

Most histories of women's health are about pre-20th century medicine and rest on limited textual evidence [9–12]. Our study, by contrast, is about modern medicine, uses text mining tools to quantify the content of thousands of clinical articles, and inferential statistics to test hypotheses. The main limitation of our study is that it is based on a single journal, one that strongly reflects British concerns. Another is that topic analysis is a rather blunt instrument for discovering what articles are about [27]. Finally, statistical patterns of historical change are, by themselves, difficult to interpret in causal terms. But they should be of interest to historians who can investigate their causes using traditional methods

Conclusions, recommendations, and future directions

In the future we hope to discover whether our findings can be generalized to the rest of the clinical literature. It would be particularly interesting to study other general medical journals. More sophisticated text-mining tools might also allow the content of articles to be explored in a more

nuanced way [64, 65]. Finally, we note that we have made our code and data public (https://github.com/Armand1/Women-in-the-BMJ-public). Many other medical subjects might be studied much as we have studied women's health, and we invite others to do so.

What is already known on this topic

- Women have been underrepresented in medical science.
- Women's health has become increasingly seen as a specific category of medicine over the years.
- Recognition of sex-specific differences, specific advances in medicine, as well as raised awareness and subsequent funding are potential driving forces.

What this study adds

- This is the first large-scale text-mining study that investigates how women have been represented in the clinical literature.
- There is a general tendency for women to become increasingly prominent in the clinical literature over time.
- The relative importance of various aspects of women's health in the clinical literature varied greatly over time driven at least in part, by contingent medical advances, public health programmes, and socio-legal changes.
- The representation of women in clinical literature, specifically whether or not articles discuss women at all, may be influenced by editorial policies.

Acknowledgements

We thank Theodora Bloom, Sean Harrop and Josie Breen of *The BMJ* for sharing article meta data, and Clare Isacke for comments on the manuscript.

Contributions

Contributors: EH, SM and AL designed the study. AB, SA and AL acquired the data. AB and AL performed the analyses. EH, JK and AL drafted the manuscript. All authors provided critical comment and feedback on the manuscript. The corresponding author attests that all listed authors meet authorship criteria and that no others meeting the criteria have been omitted. SM and AL are the guarantors of this manuscript.

Funding

This research received no funding support.

Competing Interests

All authors have completed the ICMJE uniform disclosure form at www.icmje.org/coi_disclosure.pdf and declare: no support from any organisation for the submitted work; no financial relationships with any organisations that might have an interest in the submitted work in the previous three years; no other relationships or activities that could appear to have influenced the submitted work.

Ethical approval

Not applicable.

Data sharing

We have made our code and data public: https://github.com/Armand1/Women-in-the-BMJ-public.

Transparency declaration

AL and SM (the manuscript's guarantors) affirm that the manuscript is an honest, accurate, and transparent account of the study being reported; that no important aspects of the study have been omitted; and that any discrepancies from the study as planned (and, if relevant, registered) have been explained.

Dissemination to participants and related patient and public communities

No patients were involved in setting the research question or the outcome measures, nor were they involved in the design and implementation of the study.

Legends of figures

Figure 1. Constructing a corpus of *BMJ* research articles 1948-2018.

Figure 2: The rise of "women" in *The BMJ* 1948–2018. A. The incidence of "woman/en" by year; points are mean frequencies relative to all words, error bars are 95% confidence intervals. Fitted lines are general additive and general linear models ± 1 s.e. based on publication date. The vertical grey dashed lines marks the date, 03.2005, when Fiona Godlee became editor of *The BMJ*. **B.** Predicted incidence of the 1000 most frequent words of clinical relevance, estimated by general linear models. "woman/en" is shown in pink; "man/en" in blue; all others in grey. The rate of increase of "woman/en", as estimated by the main date-of-publication effect of model fits, is faster than 81% of the other selected words. **C.** The incidence of "woman/en" by year in 20 clinical specialties defined by super-topics. The colour gradient indicates the rank order of the rate of increase; coefficient estimate (odds ± 95% C.I.) of linear models for each specialty is given in the legend. The fastest increasing super-topic is "nephrology"; "psychiatry" declines.

Figure 3: Women-specific health in *The BMJ* 1948–2018. A. The incidence of 21 aggregated women's health topics, error bars are 95% confidence intervals; light pink lines is a general additive model, dark pink line is a single-break segmented general linear model: fits±1 s.e. The vertical grey dashed lines marks the date, 03.2005, when Fiona Godlee became editor of *The BMJ*. **B.** The rise of clinical trials and the decline of case studies. Point estimates are aggregated incidence of super-topics, error bars are 95% confidence intervals; lines are general additive model fits±1 s.e.

Figure 4: Women's health topics in BMJ research articles, 1948–2018. Point estimates are aggregated incidence of super-topics, error bars are 95% confidence intervals; lines are general additive model fits±1 s.e. Points are aggregated incidence of super-topics, error bars are 95% confidence intervals; lines are general additive model fits±1 s.e. Dotted vertical lines, and labels, mark major events in medicine that likely explain some of the major changes in super-topic incidence.

Figure 5: The incidence of gender-vocal articles, 1948-2018. Error bars are 95% confidence intervals; pink line is a single-break segmented general linear model that shows a strong structural break around 2005, when the incidence rapidly increased: fits±1 s.e. The fit of this model is superior to one with no break by AIC. The red point gives the breakpoint; the error bars (not visible) are 95% confidence intervals.

References

1. Institute of Medicine. *Women's Health Research: Progress, Pitfalls, and Promise*. Washington, DC: The National Academies Press, 2010. doi:10.17226/12908
2. Van Vollenhoven RF. Sex differences in rheumatoid arthritis: more than meets the eye. *BMC medicine* 2009;7:12. doi: 10.1186/1741-7015-7-12.
3. Bleker SM, Coppens M, Middeldorp S. Sex, thrombosis and inherited thrombophilia. *Blood Reviews* 2014;3:123–133. doi: 10.1016/j.blre.2014.03.005 [published Online First: 1 April 2014].
4. Barco S, Mahmoudpour SH, Valerio L, et al. Trends in mortality related to pulmonary embolism in the European Region from 2000 to 2015: analysis of vital registration data from the World Health Organization mortality database. *Lancet Resp Med* 2020;8(3):277-28. doi: 10.1016/S2213-2600(19)30354-6 [published Online First: 12 October 2019].
5. Pinn VW. Sex and gender factors in medical studies: implications for health and clinical practice. *JAMA* 2003;289:397-400. doi: 10.1001/jama.289.4.397
6. Millett ERC, Peters SAE, Woodward, M. Sex differences in risk factors for myocardial infarction: cohort study of UK Biobank participants. *BMJ* 2018;363:k4247. doi: 10.1136/bmj.k4247
7. Legato MK, Johnson PA, Manson JE. Consideration of sex differences in medicine to improve health care and patient outcomes. *JAMA* 2016;316(18):1865–1866. doi: 10.1001/jama.2016.13995
8. Rich-Edwards JW, Kaiser UB, Chen GL, et al. Sex and gender differences research design for basic, clinical, and population studies: essentials for investigators. *Endocr Rev* 2018;39(4):424-439. doi: 10.1210/er/2017-00246
9. Sbisà M. The feminine subject and female body in discourse about childbirth. *European Journal of Women's Studies* 1996;3:363-376. doi: 10.1177/135050689600300403
10. Green MH. Gendering the history of women's healthcare. *Gender & History* 2008;20(3):487-518. doi: 10.1111/j.1468-0424.2008.00534.x
11. Shorter E. *A social history of women's encounter with health, ill-health and medicine: new edition*. Oxon, NY: Routledge, 2017.
12. Kerkhof PLM, Osto E. *Women and Men in the History of Western Cardiology: Some Notes on Their Position as Patients, Role as Investigational Study Subjects, and Impact as Professionals*. In: Kerkhof P, Miller V, eds. Sex-Specific Analysis of Cardiovascular Function. Advances in Experimental Medicine and Biology, vol 1065. Springer, Cham 2018:1-30.
13. Nichols FH. History of the women's health movement in the 20th century. *J Obstet Gynecol Neonatal Nurs* 2000;29:56-64. doi: 10.1111/j.1552-6909.2000.tb02756.x
14. Morgen S. *Into Our Own Hands: The Women's Health Movement in the United States, 1969-1990*. New Brunswick, NJ: Rutgers University Press, 2002.
15. Tuana N. The speculum of ignorance: the women's health movement and epistemologies of ignorance. *Hypatia* 2006;21(3):1-19. doi: 10.1111/j/1527-2001.2006.tb01110.x [published Online First: 9 January 2009].
16. Harvey JA, Strahilevitz MA. The power of pink: cause-related marketing and the impact on breast cancer. *J Am Coll Radiol* 2009;6(1):26-32. doi:10.1016/j/jacr.2008.07.010
17. Osuch JR, Silk K, Price C et al. A historical perspective on breast cancer activism in the United States: from education and support to partnership in scientific research. *J Womens health (Larchmt)* 2012;21(3):355-362. doi: 10.1089/jwh.2011.2862

18. Meinert CL, Gilpin AK, Unalp A, et al. Gender representation in trials. *Control Clin Trials* 2000;21(5):462-475. doi: 10.1016/s0197-2456(00)00086-6
19. Vidaver RM, Lafleur B, Tong C, et al. Women subjects in NIH-funded clinical research literature: lack of progress in both representation and analysis by sex. *J Womens Health Gend Based Med* 2000;9(5):495-504. doi:10.1089/15246090050073576
20. Geller SE, Koch A, Pellettieri B, et al. Inclusion, analysis, and reporting of sex and race/ethnicity in clinical trials: have we made progress? *J Womens Health (Larchmt)* 2011;20(3):315-320. doi: 10.1089/jwh.2010.2469 [Published Online First: 25 February 2011].
21. Liu KA, Mager NA. Women's involvement in clinical trials: historical perspective and future implications. *Pharm Pract (Granada)* 2016;14(1):708. doi: 10.18549/PharmPract.2016.01.708 [Published Online First: 15 March 2016].
22. Wallach JD, Sullivan, PG, Trepanowski JF, et al. Sex based subgroup differences in randomized controlled trials: empirical evidence from Cochrane meta-analyses. *BMJ* 2016;355:i5826. doi: 10.1136/bmj.i5826
23. Labots G, Jones A, de Visser SJ, et al. Gender differences in clinical registration trials: is there a real problem? *Br J Clin Pharmacol* 2018;84(4):700-707. doi: 10.1111/bcp.13497 [Published Online First: 12 February 2018].
24. Anderson A, McFarland D, Jurafsky, D. *Towards a Computational History of the ACL: 1980-2008*. In: Banchs RE, ed. in Proceedings of the ACL-2012 Special Workshop on Rediscovering 50 Years of Discoveries. Jeju Island, Korea: Association for Computational Linguistics 2012:13–21.
25. Shardlow M, Batista-Navarro R, Thompson P, et al. Identification of research hypotheses and new knowledge from scientific literature. *BMC Med Inform Decis Mak* 2018;18(1):46. doi: 10.1186/s12911-018-0639-1.
26. Thompson P, Batista-Navarro RT, Kontonatsios G. et al. Text mining the history of medicine. *PLoS ONE* 2016;11(1):e0144717. doi: 10.137/journal.pone.0144717.
27. Thompson P, McNaught J, Ananiadou S. *Customised OCR correction for historical medical text*. In: Guidi G, Scopigno R, Torres JC, Graf H, editors. 2015 Digital Heritage, Vol 1. Granada, Spain: IEEE 2016;25-41. doi: 10.1109/DigitalHeritage.2015.7413829.
28. Hintzen RE, Papadopoulou M, Mounce R, et al. Relationship between conservation biology and ecology shown through machine reading of 32,000 articles. *Conserv Biol* Published Online First: 8 November 2019. doi: 10.1111/cobi.13435
29. Soto AJ, Przybyła P, Ananiadou S. Thalia: semantic search engine for biomedical abstracts. *Bioinformatics* 2019;35(10):1799-1801. doi: 10.1093/bioinformatics/bty871
30. Przybyła P, Brockmeier AJ, Kontonatsios G, et al. Prioritising references for systematic reviews with RobotAnalyst: A user study. *Res Synth Methods* 2018;9(3):470-488. doi. 10.1002/jrsm.1311 [Published Online First: 30 July 2018].
31. Salton G. *The Smart document retrieval project* in Proceedings of the 14th annual international ACM SIGIR conference on Research and development in information retrieval. New York, NY: Association for Computing Machinery, 1991:356–358.
32. Blei DM, Ng AY, Jordan MI. Latent Dirichlet Allocation. *J Mach Lear Res* 2003;3(4-5):993-1022. doi: 10.1162/jmlr.2003.3.4-5.993 [Published Online First: March 2003].
33. McCallum AK. MALLET: *A machine learning for language toolkit*. http://mallet.cs.umass.edu. 2002.
34. Muggeo VM. Segmented: an R Package to Fit Regression Models with Broken-Line Relationships. *R News* 2008;8:20-25.
35. Payne RT. Oral contraceptives, thrombosis, and cyclical factors affecting veins. *BMJ* 1966;1(5490):802. doi: 10.1136/bmj.1.5480.802.

36. Meade TW, Greenberg G, Thompson SG. Progestogens and cardiovascular reactions associated with oral contraceptives and a comparison of the safety of 50- and 30-microgram oestrogen preparations. *BMJ* 1980;280(6224):1157-1161. doi: 10.1136/bmj.280.6224.1157
37. Weill A, Dalichampt M, Raguideau F, et al. Low dose oestrogen combined oral contraception and risk of pulmonary embolism, stroke, and myocardial infarction in five million French women: cohort study. *BMJ* 2016;353:i2002. doi: 10.1136/bmj.i2002
38. Van Hylckama Vlieg A, Helmerhorst FM, Vandenbroucke, JP, et al. The venous thrombotic risk of oral contraceptives, effects of oestrogen dose and progestogen type: results of the MEGA case-control study. *BMJ* 2009;339:b2921. doi: 10.1136/bmj.b2921
39. Lidegaard Ø, Løkkegaard E, Svendsen AL, et al. Hormonal contraception and risk of venous thromboembolism: national follow-up study. *BMJ* 2009;339:b2890. doi: 10.1136/bmj.b2890
40. Lidegaard Ø, Nielsen LH, Skovlund CW, et al. Risk of venous thromboembolism from use of oral contraceptives containing different progestogens and oestrogen doses: Danish cohort study, 2001-9. *BMJ* 2011;343:d6423. doi: 10.1136/bmj.d6423
41. Jick SS, Hernandez RK. Risk of non-fatal venous thromboembolism in women using oral contraceptives containing drospirenone compared with women using oral contraceptives containing levonorgestrel: case-control study using United States claims data. *BMJ* 2011;342:d2151. doi: 10.1136/bmj.d2151
42. Mantha S, Karp R, Raghavan V, et al. Assessing the risk of venous thromboembolic events in women taking progestin-only contraception: a meta-analysis. *BMJ* 2012;345:24944. doi: 10.1136/bmj.e4944
43. Vinogradova Y, Coupland C, Hippisley-Cox J. Use of combined oral contraceptives and risk of venous thromboembolism: nested case-control studies using the QResearch and CPRD databases. *BMJ* 2015;350:h2135. doi: 10.1136/bmj.h2135
44. Rodger MA, Le Gal G, Anderson DR, et al. Validating the HERDOO2 rule to guide treatment duration for women with unprovoked venous thrombosis: multinational prospective cohort management study. *BMJ* 2017;356:j1065. doi: 10.1136/bmj.j1065
45. Christin-Maitre S. History of oral contraceptive drugs and their use worldwide. *Best Pract Res Clin Endocrinol Metab* 2013;27(1):3-12. doi: 10.1016/j.beem.2012.11.004 [Published Online First: 25 December 2012].
46. Keown J. *Abortion, doctors and the law: some aspects of the legal regulation of abortion in England from 1803 to 1982* (Cambridge Studies in the History of Medicine). Cambridge, UK: Cambridge University Press 1988. doi: 10.1017/CB09780511563683
47. The Boston Women's Health Collective. *Women and their bodies.* Boston, MA: New England Free Press 1970.
48. The Lancet. Cardiology's problem women. *The Lancet* 2019;393(10175):959. doi: 10.1016/S0140-6736(19)30510-0
49. Tone A, Koziol M. (F)ailing women in psychiatry: lessons from a painful past. *CMAJ* 2018;190(20):E624-E625. doi: 10.1503/cmaj.171277 [Published Online First: 21 May 2018].
50. Riecher-Rössler A. Sex and gender differences in mental disorders. *Lancet Psychiatry* 2017;4(1):8-9. doi: 10.1016/S2215-0366(16)30348-0 [Published Online First: 15 November 2016].
51. Howard LM, Ehrlich AM, Gamlen F, et al. Gender-neutral mental health research is sex and gender biased. *Lancet Psychiatry* 2017;4(1):9-11. doi: 10.1016/S2215-0366(16)30209-7 [Published Online First: 15 November 2016].
52. Giudice LC. Endometriosis. *N Engl J Med* 2010;362(25)2389-2398. doi: 10.1056/NEJMcp1000274

53. Norman A. *Ask me about my uterus: a quest to make doctors believe in women's pain*. New York, NY: Nation Books 2018.
54. Jackson, G. *Pain and prejudice: a call to arms for women and their bodies*. London, UK: Little, Brown Book Group, 2019.
55. Ghai V, Jan H, Shakir F, et al. Diagnostic delay for superficial and deep endometriosis in the United Kingdom. *J Obstet Gynaecol* 2020;40(1):83-89. doi: 10.1080/01443615.2019.1603217 [Published Online First: 22 July 2019].
56. UK Office for National Statistics. *Domestic abuse in England and Wales: year ending March 2018*https://www.ons.gov.uk/peoplepopulationandcommunity/crimeandjustice/bulletins/domesticabuseinenglandandwales/yearendingmarch2018 (2020).
57. Smittenaar CR, Petersen KA, Stewart K, et al. Cancer incidence and mortality projections in the UK until 2035. *Br J Cancer* 2016;115(9):1147-1155. doi: 10.1038/bjc.2016.304 [Published Online First: 11 October 2016].
58. Sidhu R, Rajashekhar P, Lavin VL, et al. The gender imbalance in academic medicine: a study of female authorship in the United Kingdom. *J R Soc Med* 2009;102(8):337-342. doi: 10.1258/jrsm.2009.080378
59. Amrein K, Langmann A, Fahrleitner-Pammer A, et al. Women Underrepresented on Editorial Boards of 60 Major Medical Journals. *Gend Med* 2011;8(6):378-387. doi: 10.1016/jgenm.2011.10.007
60. Erren TC, Groß JV, Shaw DM, et al. Representation of women as authors, reviewers, editors in chief, and editorial board members at 6 general medical journals in 2010 and 2011. *JAMA Intern Med* 2014;174(4):633-635. doi: 10.1001/jamainternmed.2013.14760
61. Angell M. Shattering the Glass Ceiling. *JAMA Intern Med* 2014;174(4):635-636. doi: 10.1001/jamainternmed.2013.13918
62. Cushman M. Diversity and inclusion in a new medical journal: advancing science in the 21st century. *Res Pract Thromb Haemost* 2018;2:620–1. doi: 10.1002/rth2.12154.
63. Grinnell M, Higgins S, Yost K, et al. The proportion of male and female editors in women's health journals: A critical analysis and review of the sex gap. *Int J Womens Dermatol* 2019;6(1):7-12. doi: 10.1016/j.ijwd.2019.11.005.
64. Christopoulou F, Tran TT, Sahu SK, et al. Adverse drug events and medication relation extraction in electronic health records with ensemble deep learning methods. *J Am Med Informs Assoc* 2020;27(1):39-46. doi: 10.1093/jamia/ocz101.
65. Tsuruoka Y, Miwa M, Hamamoto K, et al. Discovering and visualizing indirect associations between biomedical concepts. *Bioinformatics* 2011;27(13):i111-119. doi: 10.1093/bioinformatics.

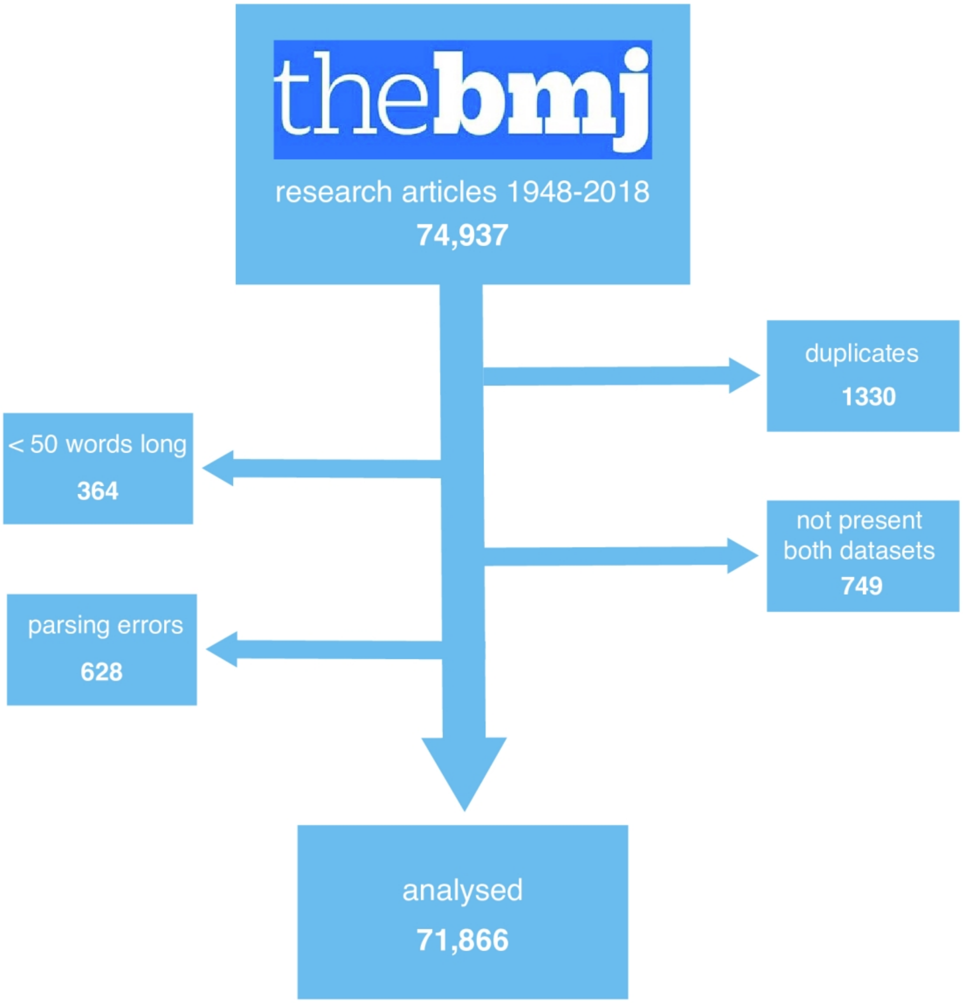

Figure 1. Constructing a corpus of *BMJ* research articles 1948-2018.

139x149mm (300 x 300 DPI)

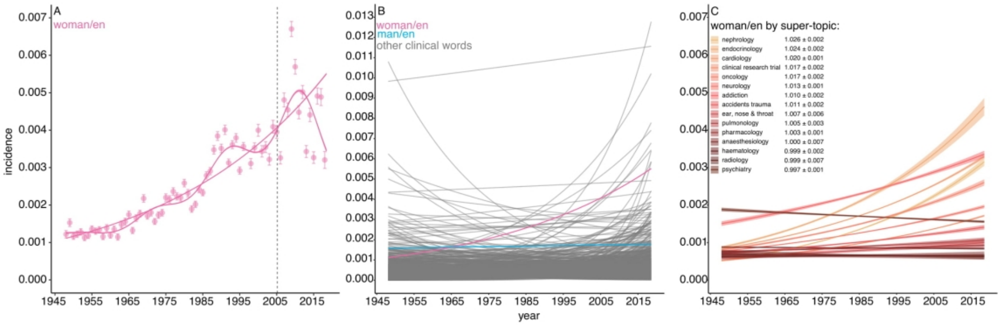

Figure 2: The rise of "women" in *The BMJ* 1948–2018. A. The incidence of "woman/en" by year; points are mean frequencies relative to all words, error bars are 95% confidence intervals. Fitted lines are general additive and general linear models ± 1 s.e. based on publication date. The vertical grey dashed lines marks the date, 03.2005, when Fiona Godlee became editor of *The BMJ*. **B**. Predicted incidence of the 1000 most frequent words of clinical relevance, estimated by general linear models. "woman/en" is shown in pink; "man/en" in blue; all others in grey. The rate of increase of "woman/en", as estimated by the main date-of-publication effect of model fits, is faster than 81% of the other selected words. **C**. The incidence of "woman/en" by year in 20 clinical specialties defined by super-topics. The colour gradient indicates the rank order of the rate of increase; coefficient estimate (odds ± 95% C.I.) of linear models for each specialty is given in the legend. The fastest increasing super-topic is "nephrology"; "psychiatry" declines.

872x285mm (72 x 72 DPI)

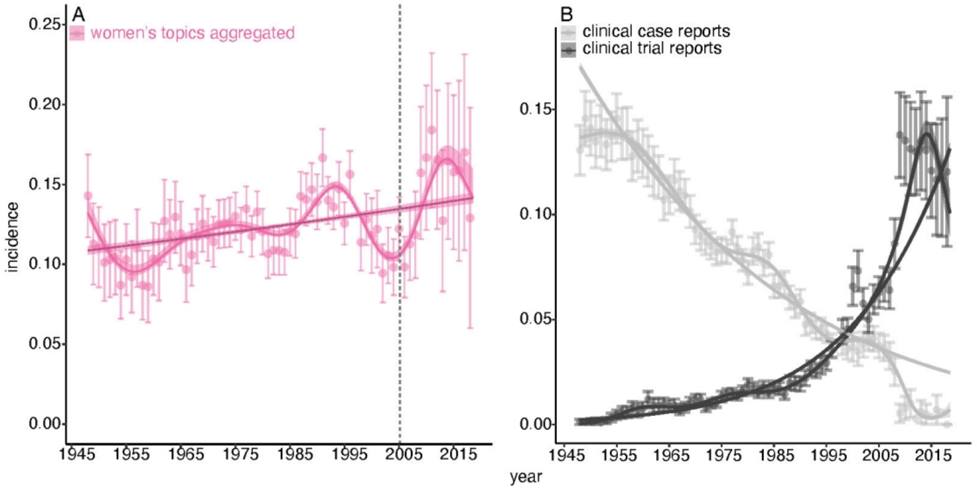

Figure 3: Women-specific health in *The BMJ* 1948–2018. A. The incidence of 21 aggregated women's health topics, error bars are 95% confidence intervals; light pink lines is a general additive model, dark pink line is a single-break segmented general linear model: fits±1 s.e. The vertical grey dashed lines marks the date, 03.2005, when Fiona Godlee became editor of *The BMJ*. **B**. The rise of clinical trials and the decline of case studies. Point estimates are aggregated incidence of super-topics, error bars are 95% confidence intervals; lines are general additive model fits±1 s.e.

209x104mm (200 x 200 DPI)

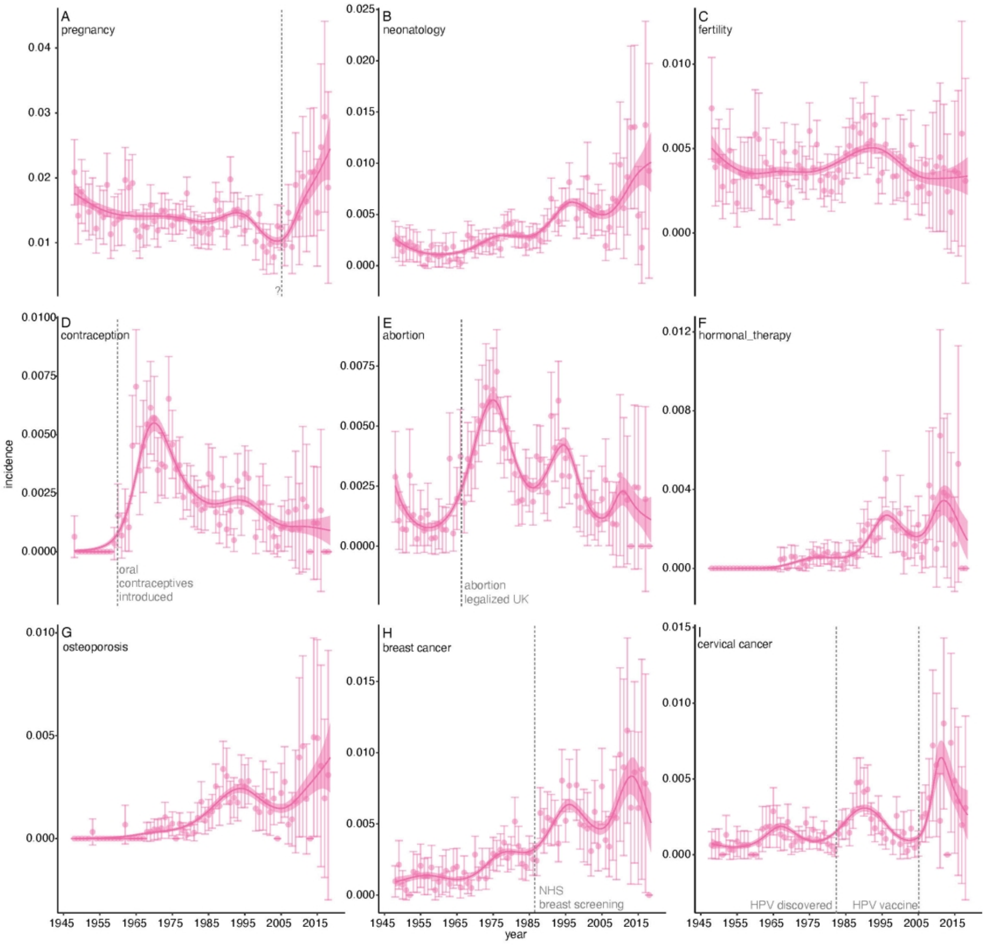

Figure 4: Women's health topics in *BMJ* research articles, 1948–2018. Point estimates are aggregated incidence of super-topics, error bars are 95% confidence intervals; lines are general additive model fits±1 s.e. Points are aggregated incidence of super-topics, error bars are 95% confidence intervals; lines are general additive model fits±1 s.e. Dotted vertical lines, and labels, mark major events in medicine that likely explain some of the major changes in super-topic incidence.

307x296mm (200 x 200 DPI)

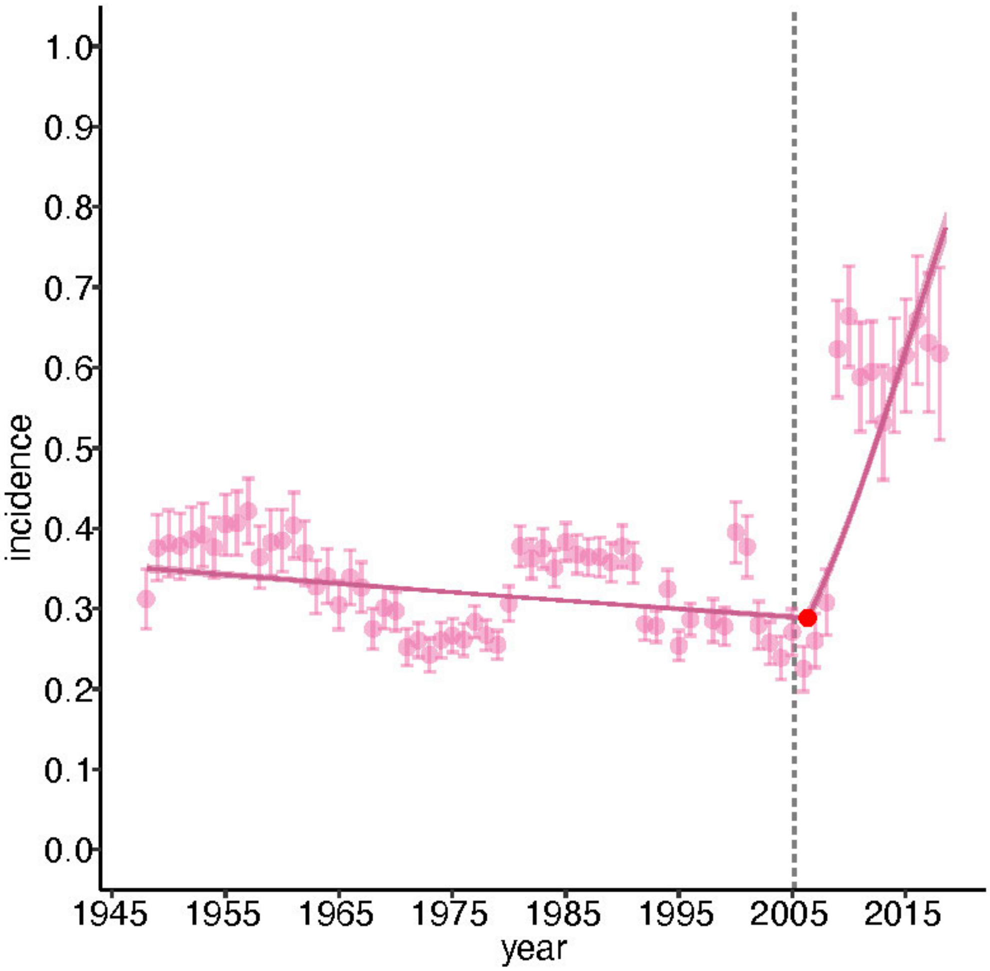

Figure 5: The incidence of gender-vocal articles, 1948-2018. Error bars are 95% confidence intervals; pink line is a single-break segmented general linear model that shows a strong structural break around 2005, when the incidence rapidly increased: fits±1 s.e. The fit of this model is superior to one with no break by AIC. The red point gives the breakpoint; the error bars (not visible) are 95% confidence intervals.

100x98mm (200 x 200 DPI)

BMJ Open

Women’s Health in *The BMJ*: A Data Science History

Journal:	*BMJ Open*
Manuscript ID	bmjopen-2020-039759.R1
Article Type:	Original research
Date Submitted by the Author:	01-Sep-2020
Complete List of Authors:	Hamulyák, Eva; Amsterdam UMC - Locatie AMC, Department of Vascular Medicine<br>Brockmeier, Austin; University of Delaware, Department of Electrical & Computer Engineering<br>Killas, Johanna; University of Toronto, Health Studies Programme<br>Ananiadou, S; The University of Manchester National Centre for Text Mining, Department of Computer Science; The Alan Turing Institute<br>Middeldorp, Saskia; Amsterdam UMC - Locatie AMC, Department of Vascular Medicine<br>Leroi, Armand; Imperial College London, Department of Life Sciences; Imperial College London, Data Science Institute
**Primary Subject Heading**:	Medical publishing and peer review
Secondary Subject Heading:	Global health, Sexual health, Research methods, Public health
Keywords:	MEDICAL HISTORY, MEDICAL JOURNALISM, STATISTICS & RESEARCH METHODS, PUBLIC HEALTH

SCHOLARONE™
Manuscripts

I, the Submitting Author has the right to grant and does grant on behalf of all authors of the Work (as defined in the below author licence), an exclusive licence and/or a non-exclusive licence for contributions from authors who are: i) UK Crown employees; ii) where BMJ has agreed a CC-BY licence shall apply, and/or iii) in accordance with the terms applicable for US Federal Government officers or employees acting as part of their official duties; on a worldwide, perpetual, irrevocable, royalty-free basis to BMJ Publishing Group Ltd ("BMJ") its licensees and where the relevant Journal is co-owned by BMJ to the co-owners of the Journal, to publish the Work in this journal and any other BMJ products and to exploit all rights, as set out in our licence.

The Submitting Author accepts and understands that any supply made under these terms is made by BMJ to the Submitting Author unless you are acting as an employee on behalf of your employer or a postgraduate student of an affiliated institution which is paying any applicable article publishing charge ("APC") for Open Access articles. Where the Submitting Author wishes to make the Work available on an Open Access basis (and intends to pay the relevant APC), the terms of reuse of such Open Access shall be governed by a Creative Commons licence – details of these licences and which Creative Commons licence will apply to this Work are set out in our licence referred to above.

Other than as permitted in any relevant BMJ Author's Self Archiving Policies, I confirm this Work has not been accepted for publication elsewhere, is not being considered for publication elsewhere and does not duplicate material already published. I confirm all authors consent to publication of this Work and authorise the granting of this licence.

For peer review only

Women's Health in *The BMJ*: A Data Science History

Eva N. Hamulyák[1*], Austin J. Brockmeier[2], Johanna D. Killas[3], Sophia Ananiadou[4,5], Saskia Middeldorp[1†], Armand M. Leroi[6,7†]

[1]*Department of Vascular Medicine, Amsterdam Cardiovascular Sciences, Amsterdam UMC, University of Amsterdam, Amsterdam, the Netherlands*
[2]*Department of Electrical & Computer Engineering, University of Delaware, Newark, Delaware, USA*
[3]*Health Studies Programme, University of Toronto, Toronto, Canada*
[4]*National Centre for Text Mining, Department of Computer Science, University of Manchester, Manchester M1 7DN, UK*
[5]*The Alan Turing Institute, London NW1 2DB, UK*
[6]*Department of Life Sciences, Imperial College London, South Kensington Campus, London SW7 2AZ, UK*
[7]*Data Science Institute, Imperial College London, South Kensington Campus, London SW7 2AZ, UK*

*Corresponding author, †joint senior author

Correspondence
Eva N. Hamulyák
E-mail: e.n.hamulyak@amsterdamumc.nl
Tel: +31 20 566 7516

Armand M. Leroi
E-mail: a.leroi@imperial.ac.uk
Tel: +44 20 7594 2396

Word count: 3943

Word count abstract: 240

Figures: 5

References: 65

Keywords: Women's health – *BMJ* – clinical studies – representation - editor

Abstract

Objective
To determine how the representation of women's health has changed in clinical studies over the course of seventy years.

Design
Observational study of 71,866 research articles published between 1948 and 2018 in *The BMJ*.

Main outcome measures
The incidence of women-specific health topics over time. General linear, additive, and segmented regression models were used to estimate trends.

Results
Over seventy years, the overall odds that a word in a *BMJ* research article was "woman" or "women" increased by an annual factor of 1.023, but this rate of increase varied by clinical specialty with some showing little or no change. The odds that an article was about some aspect of women-specific health increased much more slowly, by an annual factor of 1.004. The incidence of articles about particular areas of women-specific medicine such as pregnancy did not show a general increase, but rather fluctuated over time. The incidence of articles making any mention of women, gender or sex declined between 1948 and 2005, after which it rose steeply so that by 2018 few papers made no mention of them at all.

Conclusions
Over time women have become ever more prominent in *BMJ* research articles. However, the importance of women-specific health topics has waxed and waned as researchers responded ephemerally to medical advances, public health programmes, and socio-legal changes. The appointment of a woman editor-in-chief in 2005 may have had a dramatic effect on whether women were mentioned in research articles.

Keywords
Women's health – *BMJ* – clinical studies – representation - editor

Strengths and limitations of this study

- This is the first large-scale text-mining study that investigates how women have been represented in the clinical literature.
- Our study is about modern medicine, uses text mining tools to quantify the content of thousands of clinical articles, and inferential statistics to test hypotheses.
- Given that topic analysis is a rather blunt instrument for discovering what articles are about, there is a general tendency for women to become increasingly prominent in the clinical literature over time.
- This study is based on a single journal, *The BMJ*, one that strongly reflects British concerns.
- Statistical patterns of historical change are, by themselves, difficult to interpret in causal terms, but should be of interest to historians who can investigate their causes using traditional methods.

Introduction

Sex matters in medicine [1]. An unequal representation of women exists in leadership and medicine, and women-specific topics are often devalued [2]. From a health perspective, women and men differ in their reproductive biology, but also in the risks of many non-reproductive diseases such as auto-immune disorders and venous thromboembolism [3, 4], the relative importance of disease risk factors [5, 6], rates of diagnosis, prognoses, and how they respond to drugs [7, 8].

Compared to men, women have historically been and, in some ways still are, ill-served by medicine [5]. The deficiencies of early medicine in the treatment of women are well documented [9–12], and so are the twentieth century's campaigns for change [13–17]. The history of women's health in modern — post-1900 — clinical science has, however, been little studied. An exception is the fraught discussion over whether women have been adequately represented, or properly studied, in clinical trials [18–23]. The tendency to use men as the standard in clinical research, driven by concerns for potential teratogenic effects of drugs or by deeming women's inclusion as risker, have been suggested as explanations for the underrepresentation of women in trials [5]. Even if both sexes were included, sex disaggregation was not performed, as it is becoming standard now [21].

Clearly, sex-specific differences are present and must be considered in clinical-decision making, but the question of how women have been studied by clinical science is a much larger one than this. It includes the focus and emphasis of medical research, the communication of these findings and their translation to clinical practice, all of which have consequences for health policy [1].

Here we apply text-mining techniques [24-30] to 71,866 articles published in *The BMJ* between 1948 and 2018 in order to find out what they say about women's health. Women's health was defined as health issues relating to biological characteristics (the female sex) or the behavioural, cultural or psychological traits typically associated with the female sex (gender). Historically, the difference between the usage of these terms has been less clear. We use the results of this analysis to provide a quantitative picture of the history of women's health, as seen through the lens of one journal, over the course of seventy years.

Materials & Methods

Constructing a corpus of *BMJ* research articles

The full text *BMJ* corpus was constructed to explore the recent history of medicine [26]. The text was obtained by means of optical character recognition (OCR) on articles that were published from 1840 onwards and subsequently scanned. The meta-data and PDFs are made publicly accessible by the U.S. National Library of Medicine and PubMed Central (PMC). PMC records were used to verify the meta-data and retrieve electronic versions of full text starting from 2009. To ensure OCR quality, we used a subset of articles from 01.01.1948 to 18.07.2018 that had a DOI entry in the PMC database, were

published in *The BMJ*, including the clinical research edition, and had a publication type of "letter", "article-commentary", "review-article", or "research-article" [27]. Applying this criterion to 208,194 scanned articles and 2,151 electronic articles resulted in 74,937 articles that had been published over the course of approximately 70 years. **Figure 1** shows how these 74,937 research articles were filtered further to remove 1330 duplicate titles, 364 articles with fewer than 50 words, and 749 articles not present in both the word count and the topic probability datasets. In addition, we removed 628 articles published between 04.01.1997 and 14.02.1998 that were implausibly long, apparently due to parsing errors. The remaining 71,866 articles form the basis of our analysis.

Topic modelling

We pre-processed the corpus by removing 571 common English words, stopwords using the Smart system [31], and words which contained digits or hyphenated parts with less than one letter. To understand the content of the articles we used a text mining technique called topic modelling. The variant we used was Bayesian latent Dirichlet allocation (LDA) [32] implemented in the mallet-2.0.8 package [33]. The LDA algorithm estimates the probability of each word belonging to a topic and the probability of finding each topic in each article. This captures the likelihood of words appearing in the same document, but does not account for sentences, word order, capitalization, or punctuation. The number of topics, k, is set by the investigators; after some experimentation with $100 \leq k \leq 1000$, we settled on $k = 400$. The Bayesian hyper-parameters that control the dispersion of topics per article and words per topic were set at $\alpha = 1/300$ and $\beta = 1/100$; the algorithm optimized these every 10 epochs.

To aid the interpretation of our topics, a medical doctor (EH) labelled them based on the 20 most probable words associated with each (see online data). A second reading was done by a co-author with expertise in topic modelling (AL). Each topic was subsequently assigned to a group of related topics designated as a "super-topic". For example, the super-topic "women breast cancer" contains three subsidiary topics: breast cancer, breast cysts (associated with cancer), and breast cancer screening. Since we were only interested in clinical medicine, we ignored the topics of health care management, the clinical literature, medical profession and regions. Of the 342 remaining topics that we used, 21 were clearly about women-specific health issues, such as pregnancy, oral contraceptives and breast cancer. Other topics besides these contained the word "women" in their most-probable words, but since they also included the word "men" they were not taken to be about women's health *per se*.

Estimating the incidence of topics and words

Our LDA model produced a probability, p_{ia}, that the ith topic is found in the ath article. To simplify analysis topic probabilities were discretized, labelling a topic as present if it exceeds a threshold probability [28]. Specifically, we identified a set of topics of interest for a given analysis (e.g., all clinical topics, or all medical condition topics), standardized the topic probabilities, p_{ia} of each article by the summed probabilities of the topics in that set, and then discretized them by assuming that an article is "about" some topic if $p_{ia} \geq 0.05$. Analogously, an article is about a given super-topic if the summed probabilities of the subsidiary topics ≥ 0.05. Having obtained counts of all topics of interest in all papers, we estimated the incidence rate of a topic as:

$$I_{it} = \frac{N_{it}}{N_t}$$

where N_{it} is the number of papers published at time t that are about the ith topic, and N_t is the total number of topics of interest identified in all papers published at that time. Note that, since papers may be about multiple topics, the denominator is not the total number of papers published in a given year. In the same way, we estimate the incidence of a word as the number of times it occurs relative to the count of all words in all articles published at time t.

Constructing and analysing sub-corpora

Here we describe how we classified our articles into sub-corpora.

Articles about women-specific health topics and not

We classified articles into those that are about any aspect of women-specific health and those that are not. To do this we first filtered our dataset for the 342 clinical topics. Then, for each article, we summed probabilities of all 21 women's health-specific topics, as well the summed probabilities of all the other topics. We then standardized these two probabilities by their sum, and applied the $p_t \geq 0.05$ threshold to the standardized probabilities. These steps identified 10,158 papers about women-specific health.

Articles that are gender-vocal and gender silent

We classified articles into those that are gender vocal and gender silent. To do this we searched all articles that contained the words "woman/en" OR "female/s" OR "gender" OR "sex/es". Articles that used any of these words were labelled "gender-vocal", i.e. differences between sex or gender, male or female, man or woman were evidently discerned, and those that used none of them "gender-silent". We validated our classification by examining the full text of 100 articles — 50 chosen at random from each group — and found that nearly all gender-vocal articles indeed studied women or girls. Gender silent articles, however, may study women or girls, but just not discuss them. These steps identified 28,837 gender-vocal articles.

Statistical analysis

All analyses were done in R. To model word or topic incidence as a function of time, we used a binomial generalized linear model (GLM) implemented in base R or a generalized additive model (GAM) implemented in the mcgv package. In order to identify breaks in individual series we carried out general linear regressions using the binomial family implemented in the segmented package [34] to estimate breakpoints. In all analyses we modelled time using publication date rather than year; for clarity, however, we show estimates of yearly incidence.

Patient and public involvement

No patients were involved in setting the research question or the outcome measures, nor were they involved in the design and implementation of the study.

Results

The rise of "women" 1948-2018

To discover how the representation of women has changed over time in *The BMJ* we began by examining word counts. Considering all articles, the words "woman" or "women" appeared about 3 times in every thousand words (0.27%), however, this incidence increased from about 1 in a thousand in 1948 to about 6 in 2018 (**Figure 2A**). Using a general linear model, we estimated that the odds of a word being "woman/en" increased by an annual factor of 1.023 ± 0.0003 (estimate ± 95% C.I.); $P < 2.0 \cdot e^{-16}$, faster than 81% of the 1000 most frequent words of clinical relevance (**Figure 2B**). By contrast, the odds of a word being "man/en" increased by 1.002 ± 0.0004; $P < 2.0 \cdot e^{-16}$) — about an order of magnitude slower.

We next asked whether the representation of women increased at the same rate in the articles of all clinical specialties. In fact, it's implausible that they should. This is because some specialties embrace important women-specific health conditions whose importance has varied over time. For example, the discovery in the 1960s that oral contraceptives and, later, hormonal replacement therapy, are risk factors for venous thrombosis resulted in many studies, quite a few of which were published in *The BMJ* [35–44]. In our scheme, they ephemerally increase the representation of women in articles about "haematology".

Here, however, we sought to exclude such effects. Instead, we wanted to study the frequency of occurrence of the words "woman" and "women" in articles that could, at least in principle, be about both sexes. For this reason, we first identified and excluded all articles about women-specific health topics (see below), which left us with 62,109, the bulk of the original sample. We then classified these articles into 20 clinical specialties using our super-topics (e.g., "cardiology", 22 topics), and estimated the incidence of the words "woman/en" in each using general linear models, as above. **Figure 2C**

shows that the rate of increase of “woman/en” varied considerably among these specialties. While the odds of a word being “woman/en” increased by an annual factor of >1.02 in articles about nephrology, endocrinology and cardiology, many specialties had very low rates of increase (e.g., pharmacology) or were indistinguishable from zero (e.g, anaesthesiology). One specialty, psychiatry, actually declined.

The slow increase of women-specific health

Merely counting how often the words “woman/en” were mentioned in *BMJ* articles does not, however, tell us what they are about. Topic analysis does. Of the 400 topics discovered by the LDA model, 21were clearly about health issues specific to women. To capture whether an article is about any aspect of women-specific health we aggregated them into a single “women-specific health” super-topic. In 1948 the incidence of this supertopic was 11%; in 2018, 14%. The odds, then, that an article was about any aspect of women-specific health increased by an annual factor of 1.004 ± 0.0013; $P = 9.28 \cdot e^{-10}$ (**Figure 3A**).

This seems surprisingly slow. By way of comparison consider two other super-topics: “clinical trial reports” (8 topics), which captures articles about randomized control trials, and “clinical case reports” (5 topics), which captures case studies. The odds that an article was about the first increased by an annual factor of 1.06 ± 0.0018; $P < 2.0 \cdot e^{-16}$, and about the second decreased by an annual factor of 0.97 ± 0.0008; $P < 2.0 \cdot e^{-16}$ (**Figure 3B**). These super-topics tell the great story of 20th century clinical science: its transformation from case-based observations to testing hypotheses in groups of patients. The increase of women-specific health is a minor phenomenon compared to that — at least it is so as seen through the lens of *The BMJ.*

Why was the increase in articles about women-specific health so slow? To investigate this, we examined the subsidiary topics (**Figure 4**). For clarity we grouped them into nine super-topics: “pregnancy” (8 topics),“neonatology” (2), “fertility” (2, excluding male fertility), “contraception” (1), “abortion” (1), “hormonal therapy” (1), “osteoporosis” (1), “‘breast cancer” (3), and “cervical cancer” (2). The most common women-specific super-topic was “pregnancy” (4,744 articles); the least “hormonal therapy” (319 articles). Fits of general additive models show that the historical dynamics of these super-topics are characterized by fluctuations in incidence rather than a general increase. Not all changes are easily explained: the increase in articles about pregnancy that occurred around 2005 does not appear to be driven by any obvious medical breakthrough (**Figure 4A**). But many of the largest fluctuations are easily explained. For example, the increase in studies about contraception after 1960 is associated with the introduction of oral contraceptives [45] (**Figure 4D**). The UK’s Abortion Act of 1967 [46], engendered much discussion about that topic (**Figure 4E**). The introduction of a breast screening programme by NHS in 1988 resulted in many articles discussing its efficacy (**Figure 4H**). The 1983 discovery that the human papillomavirus’s (HPV) caused cervical cancer prompted a surge of articles about that topic, as did the implementation of an HPV vaccine in 2006 (**Figure 4I**). In general, it appears that the history of women-specific health reflects the impact of contingent events

such as medical advances, public health programmes, and socio-legal changes. Such events result in a flurry of articles which lasts for some years but then fades away as researchers turn to studying something else. The impact of the typical devaluation of topics that are feminised and the lack of women's representation in academic medicine have to be considered in this as well.

It took a woman?

Besides estimating the incidence of the words "woman/en", and the incidence of women's health-specific topics, in our articles, we also investigated how many of them mentioned women at all. To do this we classified our articles into "gender-vocal" articles that discuss women — or at least mention gender and sex differences in some way — and "gender-silent" articles that are oblivious to them. We found that in 1948 around 40% (240/606) of articles were "gender vocal", but that in 2018 84% (68/81) were (**Figure 5**). In order to confirm our results, we read the full text of many "gender silent" papers published since 2010. We found some of them did, in fact, allude to women or sex or gender differences — but only in the data tables that we removed in pre-processing. This was particularly true of dozens of articles reporting clinical trials. Although these articles may have included women in their study samples, they were truly "gender silent" in that they did not analyse or report or discuss sex-specific differences in any way.

The increase in "gender vocal" papers did not occur gradually but rather suddenly, around 2005. A segmented GLM identified a single break at 2005 ± 0.45 years (95% confidence interval), before which the odds of an article being gender-vocal had declined gradually by an annual factor of 0.99 ± 0.0008; after which it increased by annual factor of 2.18 ± 0.015. What was the cause of this dramatic change? One possibility is that it was due to a change in editorial policy. In March 2005 Fiona Godlee succeeded Kamran Abbas (acting, 2004–2005) and Richard Smith (1991–2004) to become the first female editor-in-chief in the journal's 180-year history. We estimate that an article published by her was about 31% more likely to contain the words "woman/en" than one published by her immediate predecessors: Smith/Abbas: 0.0035 ± 0.00003; Godlee: 0.0046 ± 0.00005; odds-ratio: 1.308 ± 0.2949; $P < 2 \cdot e^{-16}$. Similarly, an article published by her was 45% more likely to be gender-vocal than one published by her immediate predecessors: Smith/Abbas: 0.37 ± 0.007; Godlee: 0.54 ± 0.014; odds-ratio: 2.04 ± 0.13; $P < 2 \cdot e^{-16}$. We did not detect a difference between the editors in the probability that an article is about women's health: Smith/Abbas: 0.0361 ± 0.0012; Godlee: 0.0365 ± 0.0023; odds-ratio: 1.0134 ± 0.0758; P = 0.726). Thus, our data are consistent with the idea that Fiona Godlee had a substantial impact on the probability that a newly published article at least mentions women, but not on the probability that it considers some aspect of women-specific health.

Principal findings

We measured the representation of women in research articles in several ways. Two of these — the incidence of the words "woman/en" relative to all words (**Figure 2A**), and the incidence of gender-vocal articles (**Figure 5**) — have quite different dynamics, but concur in showing that women are now much more likely to be discussed in research articles than in the past. For at least fifty years women have asked that clinical science treat them equally to men [13–15, 47]. Clinical science, it seems, has responded — at least as seen in the pages of this journal, but the response has been uneven (**Figure 2C**). When we excluded articles about health issues specific to women, and estimated the incidence of the words "woman/en" in those that remained, we found that some — notably psychiatry — mentioned them relatively rarely; and that the rate at which they did so has not increased over seventy years. It is not that psychiatry has particularly many women-specific papers. We excluded only 9.4% (615/6595) of psychiatry articles, and our topic analysis did not identify a single women-specific psychiatric topic such as post-natal depression. This contrasts with oncology in which we excluded 21.8% (1026/4711) of articles, mostly about breast and cervical cancer; nevertheless, the rate at which women were mentioned in the remaining articles improved substantially. Cardiologists, too, appear to be addressing their "problem women" — as a *Lancet* editorial termed them [48] — but psychiatrists, it seems, must try harder [49–51]. Some are. The Royal College of Psychiatrists has had a "Women and Mental Health" special interest group since 1995.

One contributing factor is that feminised topics, such as women's health, are devalued [2]. Researchers and perhaps also the audience or *The BMJ* itself may have less interest in women's health. Another factor is that women are more likely to focus on women's health issues and subsequent research, but possibly have been underrepresented as authors for *The BMJ*. We set out to investigate this, but due to lack of bibliographic information, we were unable to assess the proportion of papers focussing on women's health to be published by female authors. Our finding that the focus on women and women's health appears to differ per discipline, could be a consequence of gender imbalance within the medical specialities. In general, gender imbalance within academic medicine and thus medical research have to be considered in interpreting these results.

Turning to articles about health issues specific to women, we found that their aggregated incidence has only increased very slightly over seventy years (**Figure 3A**). Examination of the subsidiary super-topics suggest that the dynamics of articles about women's health is driven less by a general trend towards increased representation, than by contingent medical advances, public health programmes, and socio-legal events. We make no general claims as to whether or not women-specific health is, or has been, adequately represented in the journal's pages. But we can identify some absences. Topic analysis does so since it is an unsupervised machine learning method that depends on the co-association of words among documents and, if a disease does not appear as a topic, that is probably because articles about it are rare. We have already mentioned one missing topic: post-natal

depression. Endometriosis is another — even though it afflicts 1 in 10 women in their reproductive years [52]. Its relative invisibility is confirmed by simple word searches: "pregnancy", "breast", "ovarian" appear in 366, 270, and 63 of 2305 articles published since 2008, but "endometriosis" in only 15. These results are consistent with claims that the disorder has been neglected by clinical researchers and poorly understood by physicians [53–55]. Similarly, although women are far more likely to be the victims of domestic abuse than men [56], its medical consequences did not appear as a topic in any form. By contrast, "head injuries", which is mostly about concussions in rugby players, did. Such arguments cut both ways. The UK diagnosis and mortality rates of breast and prostate cancer are nearly the same [57], but where breast cancer gets three topics ("breast cancer", "breast cysts", and "breast cancer treatment"), prostate cancer gets none. Word counts, however, show that the representation of these diseases is rapidly becoming more even (data not shown). Were we to repeat the analysis a decade hence, prostate cancer would likely have a topic of its own.

Our most intriguing result is the revelation that the incidence of gender-vocal articles increased dramatically around 2005, just when Fiona Godlee became editor-in-chief. It is easy to see how the two events might be causally connected. Perhaps authors, aware that *The BMJ* had acquired a new female editor, made sure to discuss the implications of their results in the context of women's health while previously they had been less assiduous in doing so. Or perhaps Godlee just made sure they did. Our results also suggest that her editorial intervention, if it existed, was limited, for we find no evidence that her editorship had an impact on whether or not an article was about a women-specific health topic. In order to clarify the interpretation of these results, we have written to Fiona Godlee asking her to comment on them. Additionally, in response to a lack of sex disaggregated data, some journals now require all data to be disaggregated by sex.

The effect that women editors-in-chiefs might have on the careers of other women has been much discussed [58-63]. As far as we know their effect on the articles that their journals publish has not. In the absence of direct evidence, we cannot exclude the possibility that the sudden rise of gender-vocality at the *The BMJ* is due a change in the sensibilities of its authors quite independent of its editor. Even if there is a causal association, we cannot say how general it might be. But we could, in principle, find out. Several other important medical journals —*The Lancet*, *JAMA*, *JAMA Internal Medicine*, *Annals of Internal Medicine*, and the *Cochrane Library* among others — currently have, or have had, women editors-in-chief. Given the full texts of all their articles, it would not be difficult to apply our methods to those journals — as well as to the many that have never had one.

Strengths and limitations of this study

Most histories of women's health are about pre-20th century medicine and rest on limited textual evidence [9–12]. Our study, by contrast, is about modern medicine, uses text mining tools to quantify the content of thousands of clinical articles, and inferential statistics to test hypotheses. The main limitation of our study is that it is based on a single journal, one that strongly reflects British concerns.

Another is that topic analysis is a rather blunt instrument for discovering what articles are about [27]. Finally, statistical patterns of historical change are, by themselves, difficult to interpret in causal terms. But they should be of interest to historians who can investigate their causes using traditional methods

Conclusions, recommendations, and future directions

In the future we hope to discover whether our findings can be generalized to the rest of the clinical literature. It would be particularly interesting to study other general medical journals. More sophisticated text-mining tools might also allow the content of articles to be explored in a more nuanced way [64, 65]. We strongly encourage all journals to require all data be disaggregated by sex and aim to maintain a balance in topics with attention for sex-specific differences. Finally, we note that we have made our code and data public (https://github.com/Armand1/Women-in-the-BMJ-public). Many other medical subjects might be studied much as we have studied women's health, and we invite others to do so.

Acknowledgements

We thank Theodora Bloom, Sean Harrop and Josie Breen of *The BMJ* for sharing article meta data, and Clare Isacke for comments on the manuscript.

Contributions

Contributors: EH, SM and AL designed the study. AB, SA and AL acquired the data. AB and AL performed the analyses. EH, JK and AL drafted the manuscript. All authors provided critical comment and feedback on the manuscript. The corresponding author attests that all listed authors meet authorship criteria and that no others meeting the criteria have been omitted. SM and AL are the guarantors of this manuscript.

Funding

This research received no funding support.

Competing Interests

All authors have completed the ICMJE uniform disclosure form at www.icmje.org/coi_disclosure.pdf and declare: no support from any organisation for the submitted work; no financial relationships with any organisations that might have an interest in the submitted work in the previous three years; no other relationships or activities that could appear to have influenced the submitted work.

Ethical approval

Not applicable.

Data sharing

We have made our code and data public: https://github.com/Armand1/Women-in-the-BMJ-public.

Transparency declaration

AL and SM (the manuscript's guarantors) affirm that the manuscript is an honest, accurate, and transparent account of the study being reported; that no important aspects of the study have been omitted; and that any discrepancies from the study as planned (and, if relevant, registered) have been explained.

Dissemination to participants and related patient and public communities

No patients were involved in setting the research question or the outcome measures, nor were they involved in the design and implementation of the study.

Legends of figures

Figure 1. Constructing a corpus of *BMJ* research articles 1948-2018.

Figure 2: The rise of "women" in *The BMJ* 1948–2018. A. The incidence of "woman/en" by year; points are mean frequencies relative to all words, error bars are 95% confidence intervals. Fitted lines are general additive and general linear models ± 1 s.e. based on publication date. The vertical grey dashed lines marks the date, 03.2005, when Fiona Godlee became editor of *The BMJ*. **B.** Predicted incidence of the 1000 most frequent words of clinical relevance, estimated by general linear models. "woman/en" is shown in pink; "man/en" in blue; all others in grey. The rate of increase of "woman/en", as estimated by the main date-of-publication effect of model fits, is faster than 81% of the other selected words. **C.** The incidence of "woman/en" by year in 20 clinical specialties defined by super-topics. The colour gradient indicates the rank order of the rate of increase; coefficient estimate (odds ± 95% C.I.) of linear models for each specialty is given in the legend. The fastest increasing super-topic is "nephrology"; "psychiatry" declines.

Figure 3: Women-specific health in *The BMJ* 1948–2018. A. The incidence of 21 aggregated women's health topics, error bars are 95% confidence intervals; light pink lines is a general additive model, dark pink line is a single-break segmented general linear model: fits ± 1 s.e. The vertical grey dashed lines marks the date, 03.2005, when Fiona Godlee became editor of *The BMJ*. **B.** The rise of clinical trials and the decline of case studies. Point estimates are aggregated incidence of super-topics, error bars are 95% confidence intervals; lines are general additive model fits ± 1 s.e.

Figure 4: Women's health topics in BMJ research articles, 1948–2018. Point estimates are aggregated incidence of super-topics, error bars are 95% confidence intervals; lines are general additive model fits ± 1 s.e. Points are aggregated incidence of super-topics, error bars are 95% confidence intervals; lines are general additive model fits ± 1 s.e. Dotted vertical lines, and labels, mark major events in medicine that likely explain some of the major changes in super-topic incidence.

Figure 5: The incidence of gender-vocal articles, 1948-2018. Error bars are 95% confidence intervals; pink line is a single-break segmented general linear model that shows a strong structural break around 2005, when the incidence rapidly increased: fits ± 1 s.e. The fit of this model is superior to one with no break by AIC. The red point gives the breakpoint; the error bars (not visible) are 95% confidence intervals.

References

1. Institute of Medicine. *Women's Health Research: Progress, Pitfalls, and Promise*. Washington, DC: The National Academies Press, 2010. doi:10.17226/12908
2. Criado-Perez C. *Invisible Women: Data Bias in a World Designed for Men*. New York: Abrams Press, 2019. doi.org/10.1177/0276146719875186
3. Van Vollenhoven RF. Sex differences in rheumatoid arthritis: more than meets the eye. *BMC medicine* 2009;7:12. doi: 10.1186/1741-7015-7-12.
4. Bleker SM, Coppens M, Middeldorp S. Sex, thrombosis and inherited thrombophilia. *Blood Reviews* 2014;3:123–133. doi: 10.1016/j.blre.2014.03.005 [published Online First: 1 April 2014].
5. Pinn VW. Sex and gender factors in medical studies: implications for health and clinical practice. *JAMA* 2003;289:397-400. doi: 10.1001/jama.289.4.397
6. Millett ERC, Peters SAE, Woodward, M. Sex differences in risk factors for myocardial infarction: cohort study of UK Biobank participants. *BMJ* 2018;363:k4247. doi: 10.1136/bmj.k4247
7. Legato MK, Johnson PA, Manson JE. Consideration of sex differences in medicine to improve health care and patient outcomes. *JAMA* 2016;316(18):1865–1866. doi: 10.1001/jama.2016.13995
8. Rich-Edwards JW, Kaiser UB, Chen GL, et al. Sex and gender differences research design for basic, clinical, and population studies: essentials for investigators. *Endocr Rev* 2018;39(4):424-439. doi: 10.1210/er/2017-00246
9. Sbisà M. The feminine subject and female body in discourse about childbirth. *European Journal of Women's Studies* 1996;3:363-376. doi: 10.1177/135050689600300403
10. Green MH. Gendering the history of women's healthcare. *Gender & History* 2008;20(3):487-518. doi: 10.1111/j.1468-0424.2008.00534.x
11. Shorter E. *A social history of women's encounter with health, ill-health and medicine: new edition*. Oxon, NY: Routledge, 2017.
12. Kerkhof PLM, Osto E. *Women and Men in the History of Western Cardiology: Some Notes on Their Position as Patients, Role as Investigational Study Subjects, and Impact as Professionals*. In: Kerkhof P, Miller V, eds. Sex-Specific Analysis of Cardiovascular Function. Advances in Experimental Medicine and Biology, vol 1065. Springer, Cham 2018:1-30.
13. Nichols FH. History of the women's health movement in the 20th century. *J Obstet Gynecol Neonatal Nurs* 2000;29:56-64. doi: 10.1111/j.1552-6909.2000.tb02756.x
14. Morgen S. *Into Our Own Hands: The Women's Health Movement in the United States, 1969-1990*. New Brunswick, NJ: Rutgers University Press, 2002.
15. Tuana N. The speculum of ignorance: the women's health movement and epistemologies of ignorance. *Hypatia* 2006;21(3):1-19. doi: 10.1111/j/1527-2001.2006.tb01110.x [published Online First: 9 January 2009].
16. Harvey JA, Strahilevitz MA. The power of pink: cause-related marketing and the impact on breast cancer. *J Am Coll Radiol* 2009;6(1):26-32. doi:10.1016/j/jacr.2008.07.010
17. Osuch JR, Silk K, Price C et al. A historical perspective on breast cancer activism in the United States: from education and support to partnership in scientific research. *J Womens health (Larchmt)* 2012;21(3):355-362. doi: 10.1089/jwh.2011.2862

18. Meinert CL, Gilpin AK, Unalp A, et al. Gender representation in trials. *Control Clin Trials* 2000;21(5):462-475. doi: 10.1016/s0197-2456(00)00086-6
19. Vidaver RM, Lafleur B, Tong C, et al. Women subjects in NIH-funded clinical research literature: lack of progress in both representation and analysis by sex. *J Womens Health Gend Based Med* 2000;9(5):495-504. doi:10.1089/15246090050073576
20. Geller SE, Koch A, Pellettieri B, et al. Inclusion, analysis, and reporting of sex and race/ethnicity in clinical trials: have we made progress? *J Womens Health (Larchmt)* 2011;20(3):315-320. doi: 10.1089/jwh.2010.2469 [Published Online First: 25 February 2011].
21. Liu KA, Mager NA. Women's involvement in clinical trials: historical perspective and future implications. *Pharm Pract (Granada)* 2016;14(1):708. doi: 10.18549/PharmPract.2016.01.708 [Published Online First: 15 March 2016].
22. Wallach JD, Sullivan, PG, Trepanowski JF, et al. Sex based subgroup differences in randomized controlled trials: empirical evidence from Cochrane meta-analyses. *BMJ* 2016;355:i5826. doi: 10.1136/bmj.i5826
23. Labots G, Jones A, de Visser SJ, et al. Gender differences in clinical registration trials: is there a real problem? *Br J Clin Pharmacol* 2018;84(4):700-707. doi: 10.1111/bcp.13497 [Published Online First: 12 February 2018].
24. Anderson A, McFarland D, Jurafsky, D. *Towards a Computational History of the ACL: 1980-2008*. In: Banchs RE, ed. in Proceedings of the ACL-2012 Special Workshop on Rediscovering 50 Years of Discoveries. Jeju Island, Korea: Association for Computational Linguistics 2012:13–21.
25. Shardlow M, Batista-Navarro R, Thompson P, et al. Identification of research hypotheses and new knowledge from scientific literature. *BMC Med Inform Decis Mak* 2018;18(1):46. doi: 10.1186/s12911-018-0639-1.
26. Thompson P, Batista-Navarro RT, Kontonatsios G. et al. Text mining the history of medicine. *PLoS ONE* 2016;11(1):e0144717. doi: 10.137/journal.pone.0144717.
27. Thompson P, McNaught J, Ananiadou S. *Customised OCR correction for historical medical text*. In: Guidi G, Scopigno R, Torres JC, Graf H, editors. 2015 Digital Heritage, Vol 1. Granada, Spain: IEEE 2016;25-41. doi: 10.1109/DigitalHeritage.2015.7413829.
28. Hintzen RE, Papadopoulou M, Mounce R, et al. Relationship between conservation biology and ecology shown through machine reading of 32,000 articles. *Conserv Biol* Published Online First: 8 November 2019. doi: 10.1111/cobi.13435
29. Soto AJ, Przybyła P, Ananiadou S. Thalia: semantic search engine for biomedical abstracts. *Bioinformatics* 2019;35(10):1799-1801. doi: 10.1093/bioinformatics/bty871
30. Przybyła P, Brockmeier AJ, Kontonatsios G, et al. Prioritising references for systematic reviews with RobotAnalyst: A user study. *Res Synth Methods* 2018;9(3):470-488. doi. 10.1002/jrsm.1311 [Published Online First: 30 July 2018].
31. Salton G. *The Smart document retrieval project* in Proceedings of the 14th annual international ACM SIGIR conference on Research and development in information retrieval. New York, NY: Association for Computing Machinery, 1991:356–358.
32. Blei DM, Ng AY, Jordan MI. Latent Dirichlet Allocation. *J Mach Lear Res* 2003;3(4-5):993-1022. doi: 10.1162/jmlr.2003.3.4-5.993 [Published Online First: March 2003].
33. McCallum AK. MALLET: *A machine learning for language toolkit*. http://mallet.cs.umass.edu. 2002.
34. Muggeo VM. Segmented: an R Package to Fit Regression Models with Broken-Line Relationships. *R News* 2008;8:20-25.
35. Payne RT. Oral contraceptives, thrombosis, and cyclical factors affecting veins. *BMJ* 1966;1(5490):802. doi: 10.1136/bmj.1.5480.802.

36. Meade TW, Greenberg G, Thompson SG. Progestogens and cardiovascular reactions associated with oral contraceptives and a comparison of the safety of 50- and 30-microgram oestrogen preparations. *BMJ* 1980;280(6224):1157-1161. doi: 10.1136/bmj.280.6224.1157
37. Weill A, Dalichampt M, Raguideau F, et al. Low dose oestrogen combined oral contraception and risk of pulmonary embolism, stroke, and myocardial infarction in five million French women: cohort study. *BMJ* 2016;353:i2002. doi: 10.1136/bmj.i2002
38. Van Hylckama Vlieg A, Helmerhorst FM, Vandenbroucke, JP, et al. The venous thrombotic risk of oral contraceptives, effects of oestrogen dose and progestogen type: results of the MEGA case-control study. *BMJ* 2009;339:b2921. doi: 10.1136/bmj.b2921
39. Lidegaard Ø, Løkkegaard E, Svendsen AL, et al. Hormonal contraception and risk of venous thromboembolism: national follow-up study. *BMJ* 2009;339:b2890. doi: 10.1136/bmj.b2890
40. Lidegaard Ø, Nielsen LH, Skovlund CW, et al. Risk of venous thromboembolism from use of oral contraceptives containing different progestogens and oestrogen doses: Danish cohort study, 2001-9. *BMJ* 2011;343:d6423. doi: 10.1136/bmj.d6423
41. Jick SS, Hernandez RK. Risk of non-fatal venous thromboembolism in women using oral contraceptives containing drospirenone compared with women using oral contraceptives containing levonorgestrel: case-control study using United States claims data. *BMJ* 2011;342:d2151. doi: 10.1136/bmj.d2151
42. Mantha S, Karp R, Raghavan V, et al. Assessing the risk of venous thromboembolic events in women taking progestin-only contraception: a meta-analysis. *BMJ* 2012;345:24944. doi: 10.1136/bmj.e4944
43. Vinogradova Y, Coupland C, Hippisley-Cox J. Use of combined oral contraceptives and risk of venous thromboembolism: nested case-control studies using the QResearch and CPRD databases. *BMJ* 2015;350:h2135. doi: 10.1136/bmj.h2135
44. Rodger MA, Le Gal G, Anderson DR, et al. Validating the HERDOO2 rule to guide treatment duration for women with unprovoked venous thrombosis: multinational prospective cohort management study. *BMJ* 2017;356:j1065. doi: 10.1136/bmj.j1065
45. Christin-Maitre S. History of oral contraceptive drugs and their use worldwide. *Best Pract Res Clin Endocrinol Metab* 2013;27(1):3-12. doi: 10.1016/j.beem.2012.11.004 [Published Online First: 25 December 2012].
46. Keown J. *Abortion, doctors and the law: some aspects of the legal regulation of abortion in England from 1803 to 1982* (Cambridge Studies in the History of Medicine). Cambridge, UK: Cambridge University Press 1988. doi: 10.1017/CB09780511563683
47. The Boston Women's Health Collective. *Women and their bodies.* Boston, MA: New England Free Press 1970.
48. The Lancet. Cardiology's problem women. *The Lancet* 2019;393(10175):959. doi: 10.1016/S0140-6736(19)30510-0
49. Tone A, Koziol M. (F)ailing women in psychiatry: lessons from a painful past. *CMAJ* 2018;190(20):E624-E625. doi: 10.1503/cmaj.171277 [Published Online First: 21 May 2018].
50. Riecher-Rössler A. Sex and gender differences in mental disorders. *Lancet Psychiatry* 2017;4(1):8-9. doi: 10.1016/S2215-0366(16)30348-0 [Published Online First: 15 November 2016].
51. Howard LM, Ehrlich AM, Gamlen F, et al. Gender-neutral mental health research is sex and gender biased. *Lancet Psychiatry* 2017;4(1):9-11. doi: 10.1016/S2215-0366(16)30209-7 [Published Online First: 15 November 2016].
52. Giudice LC. Endometriosis. *N Engl J Med* 2010;362(25)2389-2398. doi: 10.1056/NEJMcp1000274

53. Norman A. *Ask me about my uterus: a quest to make doctors believe in women's pain*. New York, NY: Nation Books 2018.
54. Jackson, G. *Pain and prejudice: a call to arms for women and their bodies*. London, UK: Little, Brown Book Group, 2019.
55. Ghai V, Jan H, Shakir F, et al. Diagnostic delay for superficial and deep endometriosis in the United Kingdom. *J Obstet Gynaecol* 2020;40(1):83-89. doi: 10.1080/01443615.2019.1603217 [Published Online First: 22 July 2019].
56. UK Office for National Statistics. *Domestic abuse in England and Wales: year ending March 2018*https://www.ons.gov.uk/peoplepopulationandcommunity/crimeandjustice/bulletins/domesticabuseinenglandandwales/yearendingmarch2018 (2020).
57. Smittenaar CR, Petersen KA, Stewart K, et al. Cancer incidence and mortality projections in the UK until 2035. *Br J Cancer* 2016;115(9):1147-1155. doi: 10.1038/bjc.2016.304 [Published Online First: 11 October 2016].
58. Sidhu R, Rajashekhar P, Lavin VL, et al. The gender imbalance in academic medicine: a study of female authorship in the United Kingdom. *J R Soc Med* 2009;102(8):337-342. doi: 10.1258/jrsm.2009.080378
59. Amrein K, Langmann A, Fahrleitner-Pammer A, et al. Women Underrepresented on Editorial Boards of 60 Major Medical Journals. *Gend Med* 2011;8(6):378-387. doi: 10.1016/jgenm.2011.10.007
60. Erren TC, Groß JV, Shaw DM, et al. Representation of women as authors, reviewers, editors in chief, and editorial board members at 6 general medical journals in 2010 and 2011. *JAMA Intern Med* 2014;174(4):633-635. doi: 10.1001/jamainternmed.2013.14760
61. Angell M. Shattering the Glass Ceiling. *JAMA Intern Med* 2014;174(4):635-636. doi: 10.1001/jamainternmed.2013.13918
62. Cushman M. Diversity and inclusion in a new medical journal: advancing science in the 21st century. *Res Pract Thromb Haemost* 2018;2:620–1. doi: 10.1002/rth2.12154.
63. Grinnell M, Higgins S, Yost K, et al. The proportion of male and female editors in women's health journals: A critical analysis and review of the sex gap. *Int J Womens Dermatol* 2019;6(1):7-12. doi: 10.1016/j.ijwd.2019.11.005.
64. Christopoulou F, Tran TT, Sahu SK, et al. Adverse drug events and medication relation extraction in electronic health records with ensemble deep learning methods. *J Am Med Informs Assoc* 2020;27(1):39-46. doi: 10.1093/jamia/ocz101.
65. Tsuruoka Y, Miwa M, Hamamoto K, et al. Discovering and visualizing indirect associations between biomedical concepts. *Bioinformatics* 2011;27(13):i111-119. doi: 10.1093/bioinformatics.

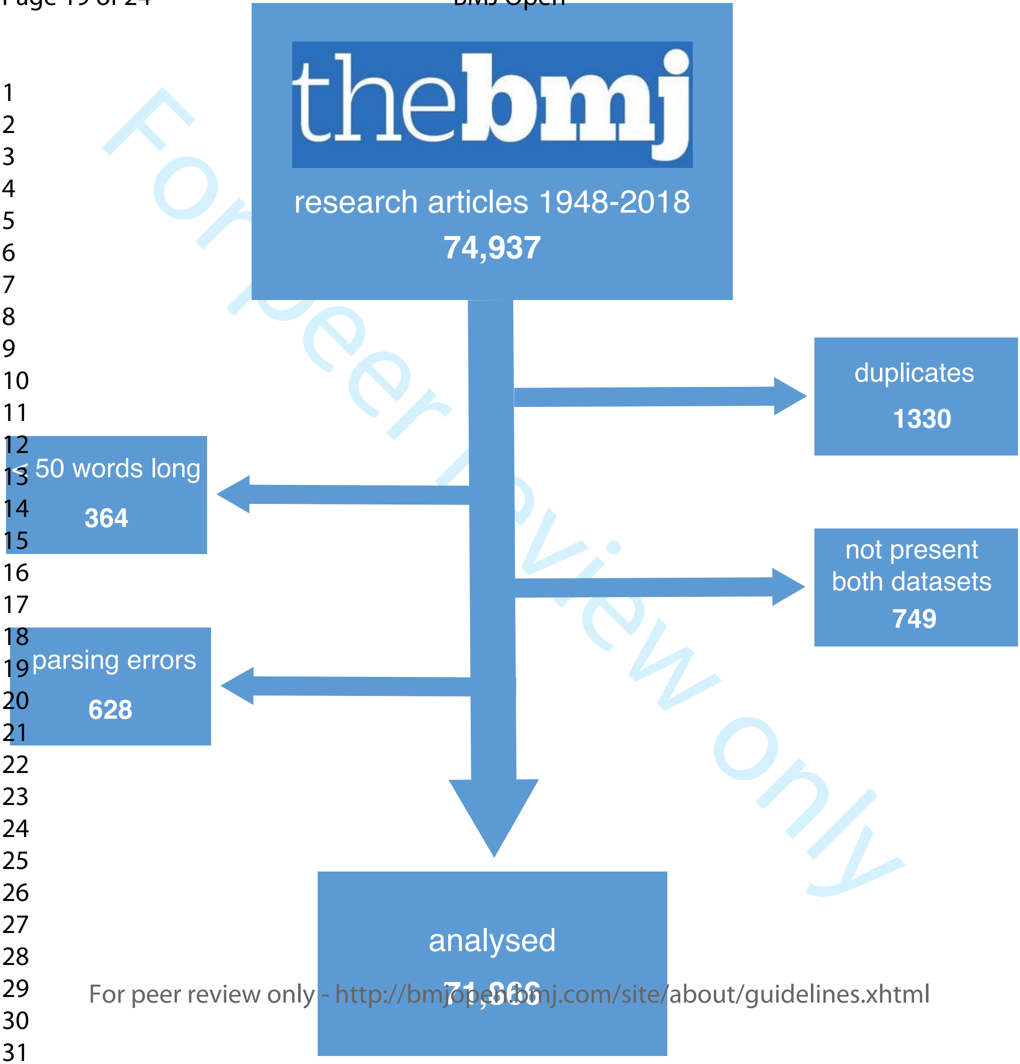

For peer review only

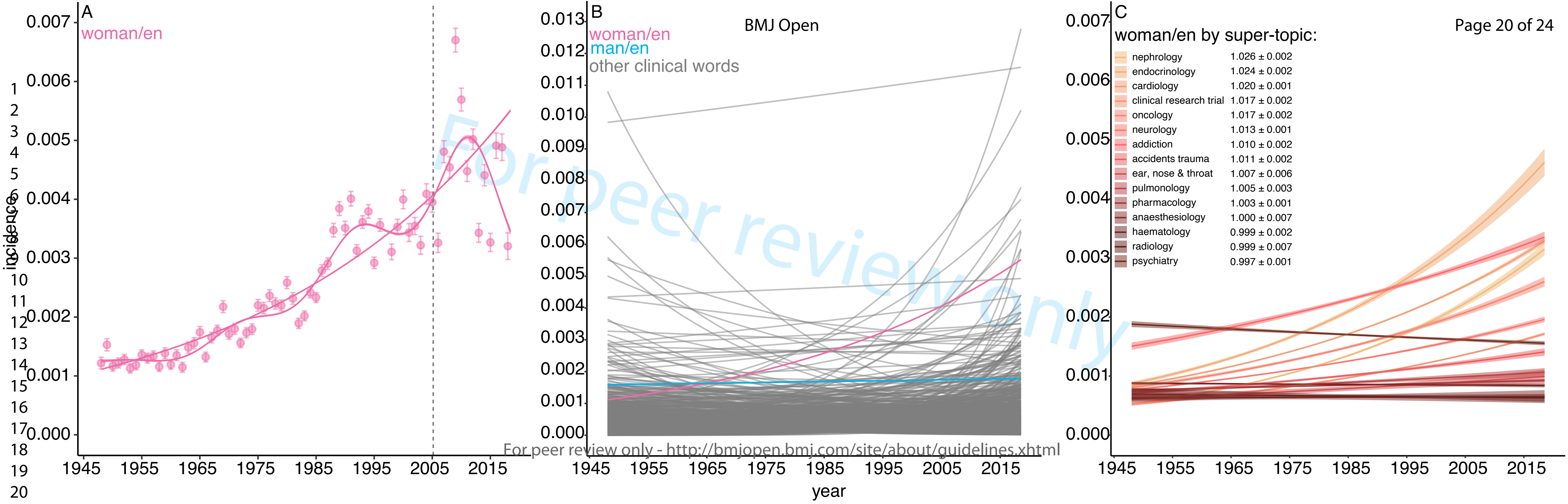

For peer review only

For peer review only - http://bmjopen.bmj.com/site/about/guidelines.xhtml

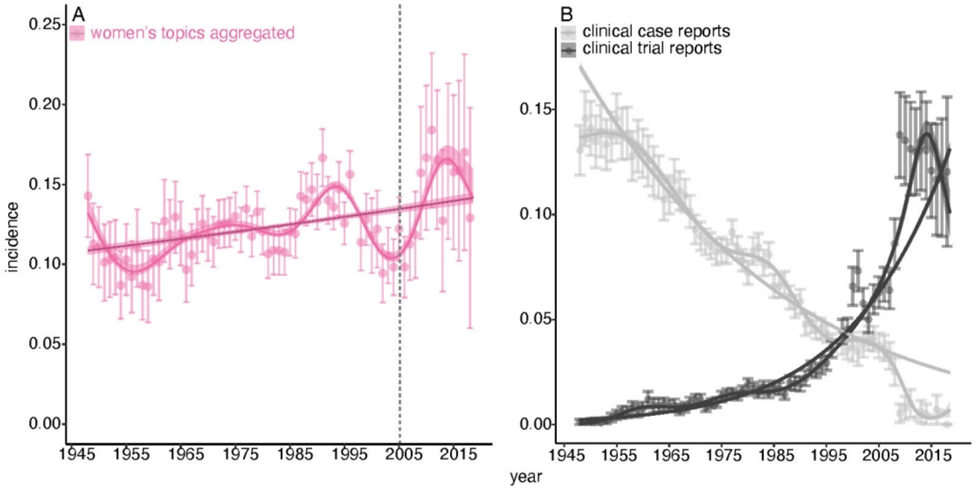

Figure 3: Women-specific health in *The BMJ* 1948–2018. A. The incidence of 21 aggregated women's health topics, error bars are 95% confidence intervals; light pink lines is a general additive model, dark pink line is a single-break segmented general linear model: fits±1 s.e. The vertical grey dashed lines marks the date, 03.2005, when Fiona Godlee became editor of *The BMJ*. **B**. The rise of clinical trials and the decline of case studies. Point estimates are aggregated incidence of super-topics, error bars are 95% confidence intervals; lines are general additive model fits±1 s.e.

209x104mm (200 x 200 DPI)

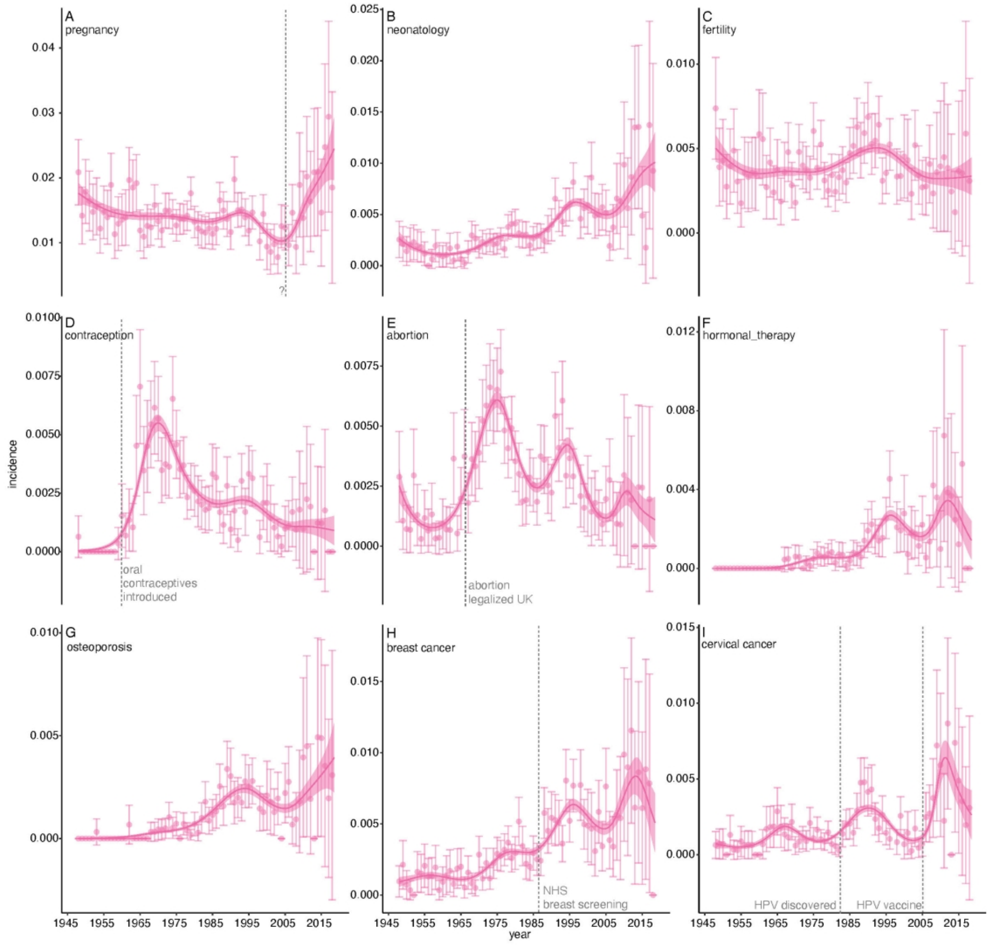

Figure 4: Women's health topics in *BMJ* research articles, 1948–2018. Point estimates are aggregated incidence of super-topics, error bars are 95% confidence intervals; lines are general additive model fits±1 s.e. Points are aggregated incidence of super-topics, error bars are 95% confidence intervals; lines are general additive model fits±1 s.e. Dotted vertical lines, and labels, mark major events in medicine that likely explain some of the major changes in super-topic incidence.

307x296mm (200 x 200 DPI)

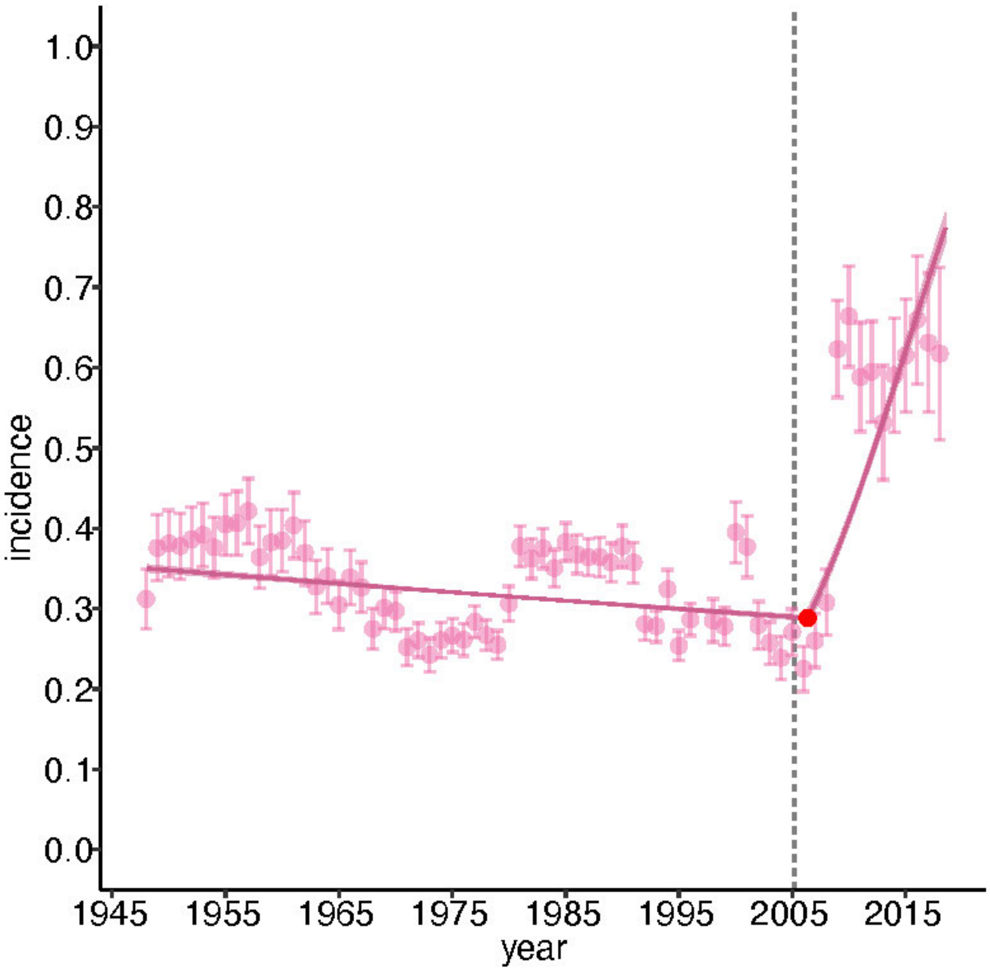

Figure 5: The incidence of gender-vocal articles, 1948-2018. Error bars are 95% confidence intervals; pink line is a single-break segmented general linear model that shows a strong structural break around 2005, when the incidence rapidly increased: fits±1 s.e. The fit of this model is superior to one with no break by AIC. The red point gives the breakpoint; the error bars (not visible) are 95% confidence intervals.

100x98mm (200 x 200 DPI)

STROBE Statement—Checklist of items that should be included in reports of ***cohort studies***

Title: Women's Health in *The BMJ*: A Data Science History

Authors: Eva N. Hamulyák, Austin J. Brockmeier, Johanna D. Killas, Sophia Ananiadou, Saskia Middeldorp, Armand M. Leroi.

Date of completing checklist: April 18th 2020

	Item No	Recommendation	Page No
**Title and abstract**	1	(*a*) Indicate the study's design with a commonly used term in the title or the abstract	1
		(*b*) Provide in the abstract an informative and balanced summary of what was done and what was found	2
**Introduction**			
Background/rationale	2	Explain the scientific background and rationale for the investigation being reported	3
Objectives	3	State specific objectives, including any prespecified hypotheses	3
**Methods**			
Study design	4	Present key elements of study design early in the paper	3
Setting	5	Describe the setting, locations, and relevant dates, including periods of recruitment, exposure, follow-up, and data collection	3
Participants	6	(*a*) Give the eligibility criteria, and the sources and methods of selection of participants. Describe methods of follow-up	3
		(*b*) For matched studies, give matching criteria and number of exposed and unexposed	
Variables	7	Clearly define all outcomes, exposures, predictors, potential confounders, and effect modifiers. Give diagnostic criteria, if applicable	3
Data sources/ measurement	8*	For each variable of interest, give sources of data and details of methods of assessment (measurement). Describe comparability of assessment methods if there is more than one group	3,4
Bias	9	Describe any efforts to address potential sources of bias	4
Study size	10	Explain how the study size was arrived at	4
Quantitative variables	11	Explain how quantitative variables were handled in the analyses. If applicable, describe which groupings were chosen and why	4,5
4,5Statistical methods	12	(*a*) Describe all statistical methods, including those used to control for confounding	5
		(*b*) Describe any methods used to examine subgroups and interactions	
		(*c*) Explain how missing data were addressed	
		(*d*) If applicable, explain how loss to follow-up was addressed	
		(*e*) Describe any sensitivity analyses	
**Results**			
Participants	13*	(a) Report numbers of individuals at each stage of study—eg numbers potentially eligible, examined for eligibility, confirmed eligible, included in the study, completing follow-up, and analysed	3-6
		(b) Give reasons for non-participation at each stage	
		(c) Consider use of a flow diagram	
Descriptive data	14*	(a) Give characteristics of study participants (eg demographic, clinical, social) and information on exposures and potential confounders	6-8
		(b) Indicate number of participants with missing data for each variable of interest	

For peer review only

For peer review only - http://bmjopen.bmj.com/site/about/guidelines.xhtml

		(c) Summarise follow-up time (eg, average and total amount)	
Outcome data	15*	Report numbers of outcome events or summary measures over time	6-8
Main results	16	(*a*) Give unadjusted estimates and, if applicable, confounder-adjusted estimates and their precision (eg, 95% confidence interval). Make clear which confounders were adjusted for and why they were included<br>(*b*) Report category boundaries when continuous variables were categorized<br>(*c*) If relevant, consider translating estimates of relative risk into absolute risk for a meaningful time period	6-8
Other analyses	17	Report other analyses done—eg analyses of subgroups and interactions, and sensitivity analyses	6-8
**Discussion**			
Key results	18	Summarise key results with reference to study objectives	9
Limitations	19	Discuss limitations of the study, taking into account sources of potential bias or imprecision. Discuss both direction and magnitude of any potential bias	10
Interpretation	20	Give a cautious overall interpretation of results considering objectives, limitations, multiplicity of analyses, results from similar studies, and other relevant evidence	9-11
Generalisability	21	Discuss the generalisability (external validity) of the study results	10
**Other information**			
Funding	22	Give the source of funding and the role of the funders for the present study and, if applicable, for the original study on which the present article is based	11

*Give information separately for exposed and unexposed groups.

Note: An Explanation and Elaboration article discusses each checklist item and gives methodological background and published examples of transparent reporting. The STROBE checklist is best used in conjunction with this article (freely available on the Web sites of PLoS Medicine at http://www.plosmedicine.org/, Annals of Internal Medicine at http://www.annals.org/, and Epidemiology at http://www.epidem.com/). Information on the STROBE Initiative is available at http://www.strobe-statement.org.

For peer review only
